# Comparative analysis of commercial human primary mesangial cell, implications for experimental design

**DOI:** 10.1186/s12882-025-04444-1

**Published:** 2025-09-29

**Authors:** Alva Johansson, Gayathri Narasimhan, Katharina Keuenhof, Roberto Boi, Kerstin Ebefors

**Affiliations:** Institute of Neuroscience and Physiology, Department of Physiology, Sahlgrenska Academy, University of Gothenburg, Box 432, Gothenburg, 40530 Sweden

**Keywords:** Mesangial cells, Proliferation, Proteomics, Cell culture

## Abstract

**Background:**

Mesangial cells (MCs) are involved in several glomerular diseases such as IgA nephropathy and diabetic kidney disease. In vitro work on human MCs is mainly conducted on primary MCs. However, cells from different donors could be significantly different, thus potentially affecting the outcome of the experiments.

**Method:**

We have compared commercially available primary human MCs from two different sources: HMCv1 and HMCv2. The cells were characterized using qPCR, western blot, and immunofluorescence. Response to PDGF-BB was assessed with proliferation assays, proteomics, and qPCR. Response to angiotensin II was assessed through contractility assay and response to IL-1β, diabetic milieu and TGFβ1 with qPCR.

**Results:**

Cells from both sources expressed mesangial markers. HMCv1, but not HMCv2, showed significant contractility in response to angiotensin II. Both HMCv1 and 2 significantly increased their proliferation rate in response to PDGF-BB. Proteomics revealed a stronger response to PDGF-BB for HMCv1 in respect to HMCv2, though similar pathways were regulated in both. IL-1β stimulus was stronger in HMCv1 in terms of increased expression of IL6 and CCL2/MCP1 mRNA. Diabetic milieu increased expression of IL-6 for both HMCv1 and 2, but significantly higher for HMCv1. TGFβ1 gave similar results in terms of IL-6 expression for cells from both sources. In addition, a list of 144 potential mesangial markers was compiled, that can be used for identification of MCs in omics data.

**Conclusion:**

This study shows that there are broad differences between sources of primary human MCs. The potential differences between clones of primary MCs need to be carefully considered when conducting in vitro experiments.

**Supplementary Information:**

The online version contains supplementary material available at 10.1186/s12882-025-04444-1.

## Background

The glomerulus is the filtration unit of the kidney, responsible for constantly filtering blood and producing primary urine. Several of the most common kidney diseases start in the glomerulus and involve different types of cells resident in these units. Podocytes (specialized epithelial cells) and glomerular endothelial cells make up the filtration barrier, residing on opposite sides of the glomerular basement membrane. The third cell type, the mesangial cells (MCs), is not part of the filtration barrier but forms the central structural stalk of the glomerulus [1]. The MCs are central to some of the most common glomerular diseases such as IgA nephropathy (IgAN) [2] and diabetic kidney disease (DKD) [3] and are therefore important when studying the onset and molecular mechanisms of these diseases.

The MCs are found in between the capillaries in the glomeruli and are embedded in their own matrix. In disease state, they are known to proliferate, become hypertrophic and expand their matrix. This results in reduced filtration area and loss of glomerular function [4]. MCs are not only important for the structure of the glomerulus, but they release and react to a multitude of growth factors, cytokines and other signaling molecules as well. For these reasons it is believed that they are central for glomerular crosstalk [4]. Platelet derived growth factor (PDGF) is the main growth factor for mesangial proliferation [5], while transforming growth factor beta 1 has been described as the one that mostly influences matrix expansion [6, 7] and hypertrophy [8]. Studying intricate cell signaling pathways in the MCs in vivo comes with challenges as the cells lack specific genes that can be used for cell-specific knock outs in animals [9]. Therefore, cell signaling events in the MCs are commonly studied in vitro. Drawbacks of relying only on the in vitro models include changes in phenotype, de-differentiation as well as the lack of crosstalk with the other cells normally found in the glomerulus.

Primary MCs can be cultured from glomeruli and are commercially available. Using primary cells for in vitro experiments is usually considered the best option, but over the years we have observed that primary human MCs (HMCs) from different donors and sources behave differently, and we speculate that there might be differences in their response and physiology due to their different origin and propagation methods.

The aim of this research was to compare two commercially available HMCs from two different sources to elucidate similarities and differences. Diverse techniques were used to characterize the cells and their response to angiotensin II, IL-1β and PDGF-BB.

## Methods

### Primary human mesangial cells

HMCs were purchased from two different vendors, Cell systems (HMCv1) and Novabiosis (HMCv2). A proteomic dataset made with a third mesangial cell type, HMCv3 (Lonza), was used for comparison purposes [10]. The cells were provided cryopreserved at passage three on arrival. HMCv1 was isolated from an adult male, and HMCv2 from an adult female. HMCv1 was characterized by the vendor through positive expression of cytoplasmic vimentin intermediate filaments, cytoplasmic alpha-actinin microfilaments and cytoplasmic fibronectin and negative for E-selectin. HMCv2 was characterized by the vendor through the expression of PDGFRB, vimentin and smooth muscle actin.

### Cell culture of primary mesangial cells

Cells were expanded from passage 3 according to the vendors’ instructions. Cells at higher passage were then cultured in DMEM/F12 medium (Gibco) supplemented with 10% FBS (Hyclone) and 1% penicillin/streptomycin (Gibco). Before the experiments cells were starved overnight in medium containing 0.5% FBS.

### Quantitative PCR

Cells were cultured as stated above and then harvested for gene expression measurements. Both cell types were cultured to the same confluency (~ 80%), washed with PBS and then harvested using RLT buffer (Qiagen). RNA was purified using the mini kit (Qiagen) and transformed to cDNA with the High-capacity RNA-to-cDNA kit (Thermo Fisher). qPCR was performed using the Quantstudio 7 pro system (Applied Biosystems) using TaqMan probes (Thermo Fisher) for ACTA2, PDGFRA, PDGFRB, CNN1 GATA3, IL6, TNFα and CCL2/MCP1. GAPDH was used as housekeeping gene.

### Staining and visualization

The cellular phenotype was observed during cell culture and visualized using an Eclipse TS100 microscope (Nikon) equipped with an Axiocam 305 color camera (Zeiss). For immunofluorescence staining cells were fixed with 4% paraformaldehyde in PBS for 10 min before being washed with PBS and blocked (PBS with 2% BSA, 2% FBS and 0.2% fish gelatin) for 30 min. Anti-smooth muscle actin (Proteintech, 14395-1-AP, 1:500) was used as primary antibody and incubated for 1 h in room temperature. After washing with PBS, the secondary antibody, anti-rabbit Alexa Fluor 488 (1:2000), was added and incubated for 1 h in room temperature before final wash and mounting using ProLong Glass Antifade Mountant with NucBlue (Invitrogen). Staining was visualized with Zeiss confocal microscope LSM 800 (Zeiss).

### Protein expression through western blot

HMCv1 and 2 were cultured as described above and harvested in Triton X-100 1% v/v, Tris-HCl 50 mM, NaCl 150 mM, pH 7.5 supplemented with Complete (Roche) protease inhibitor tablets and PhosSTOP tablets (Roche). Mesangial cell marker analysis was performed using mini protean TGX Stain-Free gels 4–15% (Bio-Rad). Proteins were transferred onto a low fluorescence PVDF membrane using the Trans-Blot Turbo Transfer system (Bio-Rad). The membranes were incubated with primary antibodies diluted in 20 mM tris-hydroxymethyl aminomethane, 1% tween 20 (TBST, Sigma-Aldrich), pH 8.0 containing 5% milk blocking buffer (Carl Roth) and incubated overnight at 4 °C. A secondary anti-rabbit (Promega, W4011) or anti-mouse HRP-conjugate (Promega, W4021) antibody was added and incubated for 1 h at room temperature (1:10000). Protein bands were developed using Clarity Western ECL solutions (Bio-Rad Laboratories) in ChemiDoc Imager (Bio-Rad). For quantification of proteins either GAPDH (Santa Cruz Biotechnology, 32233) or the total protein blot (stain free normalization method, Bio-Rad) [11] was used to normalize between groups. The following primary antibodies were used: anti-αSMA (Proteintech, #14395-1-AP), anti-PDGFRα (Cell Signaling Technology, #3174T), anti-PDGFRβ (Cell Signaling Technology, 3169T), anti-PDGFRbeta phospho Y1009 (Cell Signaling Technology, #3124S) and anti-PDGFRbeta phospho Y751 (Cell Signaling Technology, #3161S). All primary antibodies were used at 1:1000 v/v dilution in TBST in 5% powdered milk (Carl Roth), with an overnight incubation at 4 °C.

### Quantitative mass spectrometry

Cells were cultured as above and starved overnight. Cells were either left unstimulated or stimulated with 25ng/ml of PDGF-BB (RnD systems) for 24 h before harvesting in 2% sodium dodecyl sulfate and 50mM triethylammonium bicarbonate (TEAB). For the complete proteomics method (digestion, fractionation, protein identification and relative quantification), please refer to the supplementary materials. Four replicates per condition (untreated vs. PDGF-BB treated, HMCv1 and HMCv2) were submitted for proteomics preparation (a total of 16 samples).

Proteomic analysis was performed with Qlucore Omics Explorer 3.9 (Qlucore, Lund, Sweden). Qlucore GSEA, Reactome (v86, 09.2023), Metascape (v3.5, http://metascape.org) and Cytoscape (v3.10) software were used for pathway analysis. Venn diagrams were made with Qlucore and the tool at https://bioinformatics.psb.ugent.be/webtools/Venn.

Differentially expressed proteins were identified using a two-sample t-test and the difference expressed as fold change (FC) or Log2FC. Significant differences were defined by a *P* < 0.05 and FC ± 20%. Distribution analysis and principal component analysis (PCA) were used as quality control for the samples and to validate clustering of the untreated and treated cells. Finally, only regulated pathways with Q < 0.05 were considered in the pathway analysis, unless otherwise stated.

A list of human glomerular MCs markers was compiled based upon 12 recent (2017–2023) articles containing omics data [10, 12–22]. Markers were ranked based upon the number of reports and had all a minimum of 2 reports in the literature mined. Markers list, tracking and references are reported in Supplementary Table 1. For the analysis of the components of the PDGF signaling pathway we used the list of proteins in the R-HSA-186797 Reactome pathway (https://reactome.org/content/detail/R-HSA-186797).

### Cell proliferation

Cells were seeded in a 96 well plate (5000 cells/well) and then starved overnight. PDGF-BB (25ng/ml) (RnD systems) was used to stimulate proliferation. Proliferation was measured as the incorporation of BrdU (Cell proliferation ELISA, BrdU, chemiluminescent, Roche) according to the manufacturer’s instructions. Three independent experiments were conducted per each cell type.

### Cell contractility

HMCv1 and 2, were seeded into 12 well glass bottom plates (50000 cells/well). Before the experiments, the cells were starved overnight. The medium was then removed and new starvation medium with 1µM angiotensin II (Merck) was added to cells. Cells were imaged over time (at 37 °C with 5% CO_2_) from 3 min to 20 min after angiotensin II addition, at 40X magnification using Cell Discoverer 7 (Zeiss). Contraction at two different timepoints was measured using the distance measuring tool “distance tool” under the “custom graphics” tab in the program Zen blue 3.4 (Zeiss). The width of the cells was measured in the same area of each cell (cell width, across the cells’ nuclei). Measurement was done on 15 random cells from each source and presented as relative change of each cell in percentage. Width differences were calculated using the following formula:

$$\frac{((\text{Width}\:20\:\text{minutes}\:\mu\text{M}-(\text{Width}\:3\:\text{minutes}\:\mu\text{M}))}{(\text{Width}\:3\:\text{minutes}\:\mu\text{M})^*{100}}$$

### Inflammatory response

HMCv1 and 2 were cultured as described above and starved overnight before treatment with either 1nM of IL-1β (interleukin 1 beta, RnD systems) for 1–6 h, 25mM L-glucose (Sigma-Aldrich) and 17µM HSA (human serum albumin, Sigma-Aldrich) (osmotic control), 25mM D-glucose (Sigma-Aldrich) and 100µM PA (palmitic acid, Sigma-Aldrich) (diabetic milieu), or 25ng/ml TGFβ1 (transforming growth factor beta 1, Bio-Techne) for 24 h. Cells were washed twice with PBS and harvested in RLT buffer (Qiagen). Gene expression of IL6 (interleukin 6), TNFα (tumor necrosis factor alpha) and CCL2/MCP1 (chemokine C-C motif ligand 2 or monocyte chemoattractant protein-1) was measured with qPCR and compared to untreated controls.

### Statistics

Statistics were performed using the GraphPad Prism software (version 9). Groups were tested for normal distribution using the Shapiro-Wilk test. For the proliferation assay, treated and untreated groups were tested using a two-tailed Mann-Whitney test. Contractility assays, Mann-Whitney test was used. Proteomic statistics are presented in the proteomic method section.

## Results

### Cell characteristics of HMCv1 and HMCv2

**Fig. 1:**
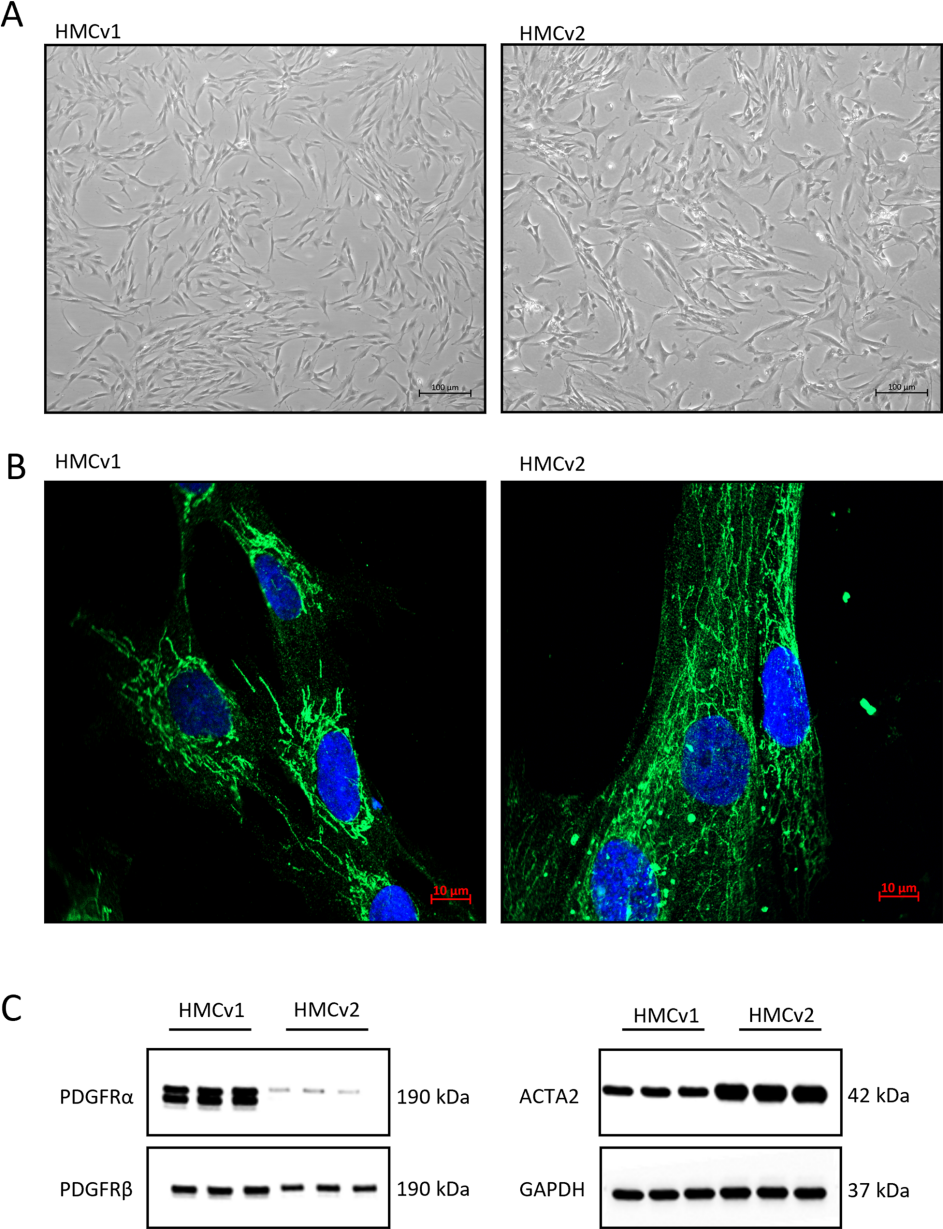
Characterization of HMCv1 and HMCv2. Morphology of HMCv1 and 2 in culture (**A**). Representative images of immunofluorescence staining of smooth muscle actin (green) and nuclei (blue) in HMCv1 and 2 (**B**). Protein expression of mesangial markers PDGFRA, PDGFRB and ACTA2 by western blotting. GAPDH was used as housekeeping protein (**C**). The uncut blots are provided in the supplemental materials. *n* = 3 replicates per cell type and treatment

MCs in vitro are usually described as characterized by a stellate shape and the ability to grow “in hillocks and valleys” [23]. The phenotype of the HMCs from the two sources differed, with HMCv1s being more elongated and growing in a more swirl-like pattern compared to the HMCv2s (Fig. 1A). Conversely, HMCv2s had a more stellate phenotype and a slower growth pace. Both HMCv1 and HMCv2 expressed smooth muscle actin (ACTA2), a marker for cultured MCs (Fig. 1B). Furthermore, HMCs from both sources expressed the mesangial marker genes ACTA2, PDGFRA (platelet derived growth factor receptor alpha), PDGFRB (beta), GATA3 (G-A-T-A containing sequence binding protein 3), and CNN1 (calponin 1) at the transcriptomic level (Table 1). The expression of PDGFRA, PDGFRB, and ACTA2 was confirmed with western blot (Fig. 1C, supplementary Fig. 1).

**Table 1:** Delta Ct values for mesangial markers

	ΔCT	
Gene	HMCv1	HMCv2
ACTA2	3.3	0.3
PDGFRA	1.7	5.8
PDGFRB	2.0	4.3
GATA3	11.9	6.2
CNN1	5.3	3.8

GAPDH was used as housekeeping gene

### Mass spectrometry, mesangial and other glomerular cell markers

**Fig. 2:**
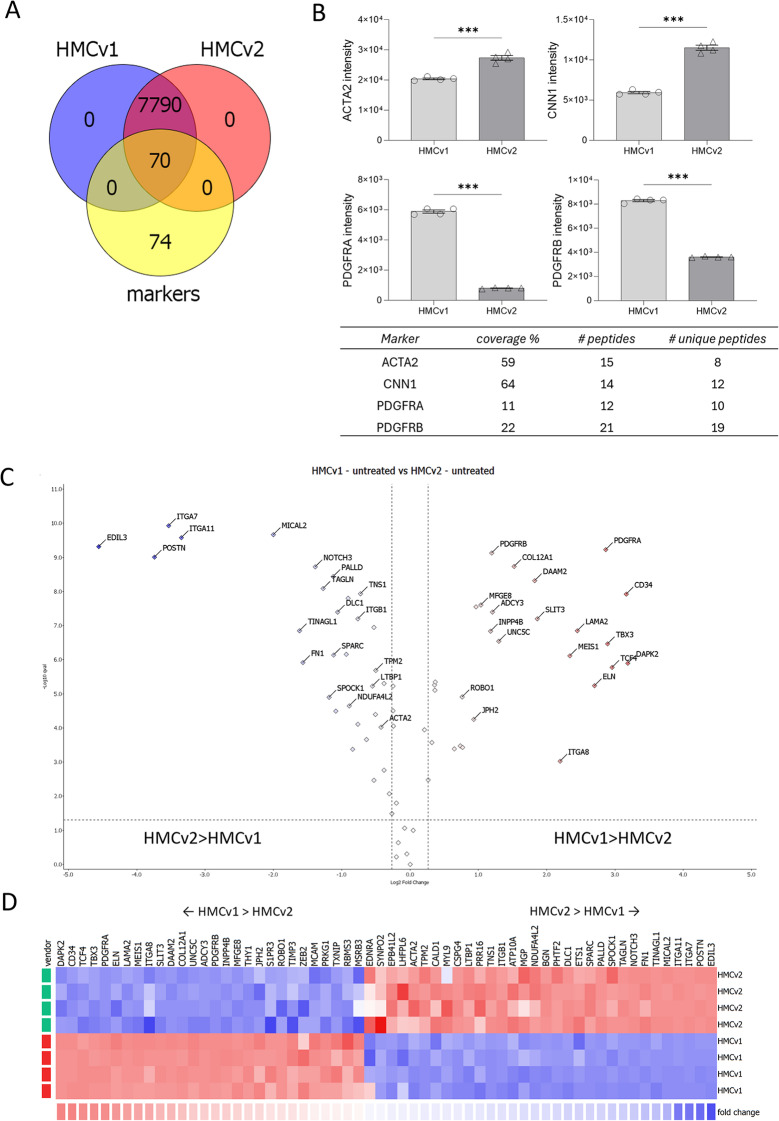
Expression of mesangial markers in HMCv1 and HMCv2. Among the 7860 identified and quantified proteins, both HMCv1 and HMCv2 cells expressed 70 out of the 144 mesangial cell markers list we compiled from recent literature (2017–2023), with focus on recent transcriptomics and single cells analysis (**A**). Venn diagram describing the overlap of markers in HMCv1 and 2 (**B**). Intensity, coverage and peptides identified for a selection of mesangial markers (**C**). Volcano plot comparing the mesangial marker protein expression in HMCv1 and 2. The dotted lines represent 0.05 p-value and ± 20% fold change thresholds, but all proteins detected are plotted (**D**). Heatmap comparing the expression of mesangial markers in HMCv1 and v2 (fold change range scaled from − 2 to + 2, 0.05 q-value and ± 20% fold change thresholds are applied). *n* = 4 replicates per treatment, cell type

The proteomics analysis identified 8297 proteins. Of those, 7860 proteins were quantified in cells from both vendors, while 423 proteins could not be quantified due to their low levels. 14 proteins were excluded from the quantification due to missing values. The expression of 144 mesangial markers was investigated in the proteomic datasets of unstimulated HMCv1 and 2. The list of mesangial markers was compiled based upon recent literature (2017–2023), with special focus on recent transcriptomics and single cells analysis (Supplemental Table 1). Markers identified in at least two different publications were used for this comparison. Of the compiled markers, 70 were found in both HMCv1 and HMCv2 (Fig. 2A). Five mesangial markers (ACTA2, CNN1, PDGFRA, PDGFRB and GATA3, already identified with western blot or qPCR) were specifically searched in the dataset. All were found, except GATA3. ACTA2 and CNN1 had the highest intensity in the HMCv2 (*P* < 0.001 for both), while PDGFRA and PDGFRB had significantly higher intensities in HMCv1 (*P* < 0.001 for both) (Fig. 2B). The extent of expression of all 70 MCs markers were similar between the cells from the two sources. The intensity of 28 markers was higher in HMCv1 and 32 markers had a higher intensity in HMCv2 cells instead (Fig. 2C-D).

The expression of glomerular endothelial and podocyte markers in the HMCs was investigated as well. The endothelial marker coagulation factor VIII (F8) was found in both HMCv1 and HMCv2, but the sequence coverage was low (1% and only 2 unique peptides were identified), while CD31 (PECAM) was not found. The podocyte marker synaptopodin (SYNPO) was found in both HMCv1 and HMCv2 with a small but significant higher intensity in HMCv2 than 1 (*P* < 0.01). The podocyte markers nephrin (NPHS1) and podocin (NPHS2) were not found (Supplementary Fig. 2).

### HMCv1 and HMCv2 proliferate when stimulated with PDGF-BB

**Fig. 3:**
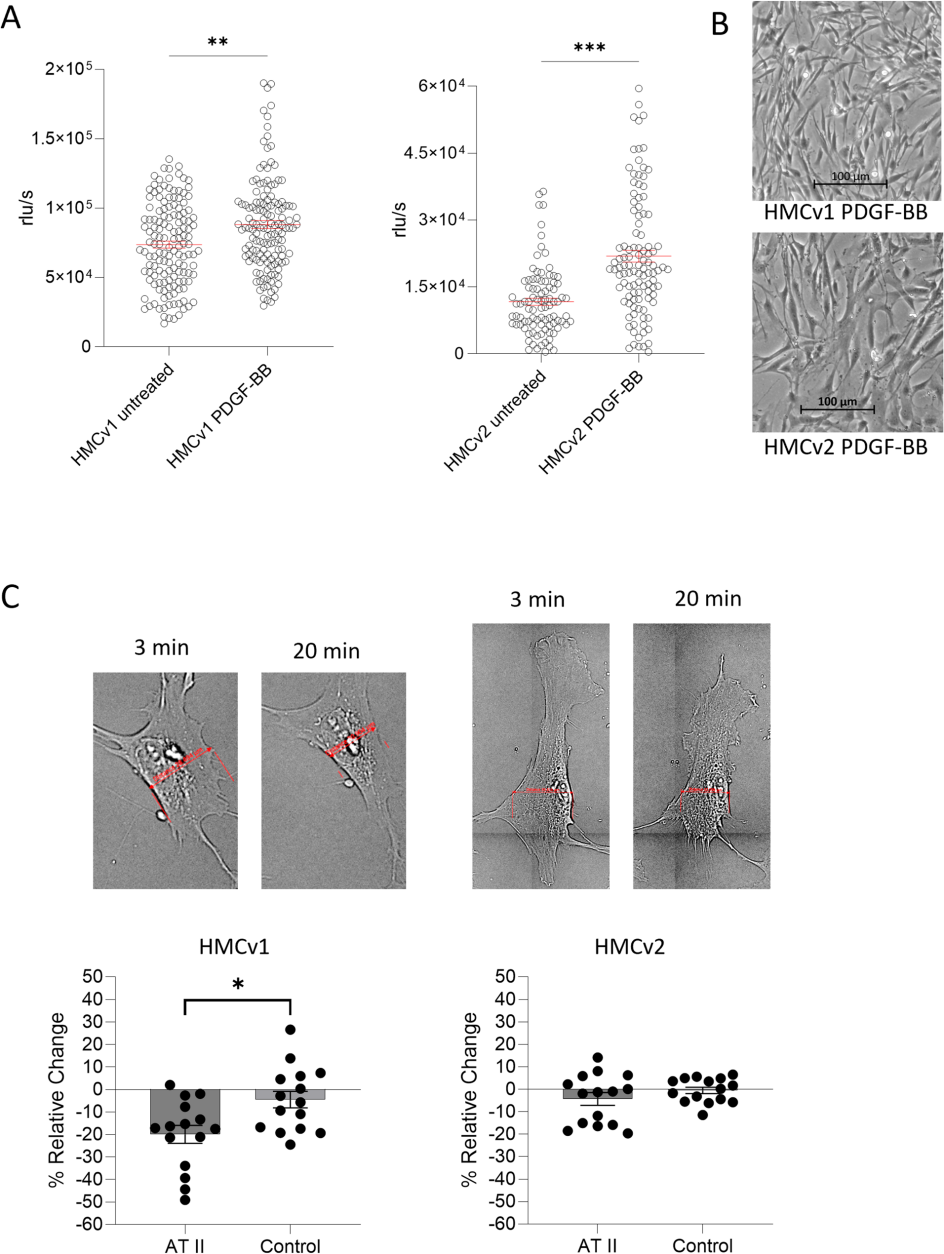
Mesangial response to PDGF-BB and angiotensin II stimulation. Proliferative response of HMCv1 and 2 after stimulation with 25 ng/ml of PDGF-BB for 24 h. Data from 3 independent experiments is shown (**A**). Representative pictures of cell morphology of HMCv1 and 2 after stimulation with 25 ng/ml of PDGF-BB for 24 h (**B**). Representative pictures showing the contraction of HMCv1 and HMCv2 after stimulation with 1µM of angiotensin II (**C**). Images were taken after 3 and 20 min and the graphs show the difference in cell width from measurements of *n* = 15 cells from each vendor. Error bars represent SEM, * *P* < 0.05, ** *P* < 0.01, *** *P* < 0.001. Proliferation and contractility assay: Mann-Whitney test

PDGF-BB is a well-known stimulator of mesangial proliferation. As expected, HMCs from both sources increased their proliferation in response to PDGF-BB compared to unstimulated cells (HMCv1, *P* < 0.01; HMCv2, *P* < 0.001), Fig. 3A. Both HMCv1 and HMCv2 became more elongated when stimulated with PDGF-BB, indicating a proliferative/migratory phenotype (Fig. 3B).

### HMCv1 show a more contractile phenotype than HMCv2

MCs in vitro are known to contract in response to angiotensin II [24]. HMCv1 responded to angiotensin II stimulation with significant contraction (*P* < 0.05). HMCv2 had a lower response to the stimulation and did not reach the significance threshold (Fig. 3C).

### Proteomics of PDGF-BB stimulated mesangial cells

To get further insight into the signaling of the cells in response to PDGF-BB, quantitative mass spectrometry was performed on stimulated and unstimulated cells from both sources.

**Fig. 4:**
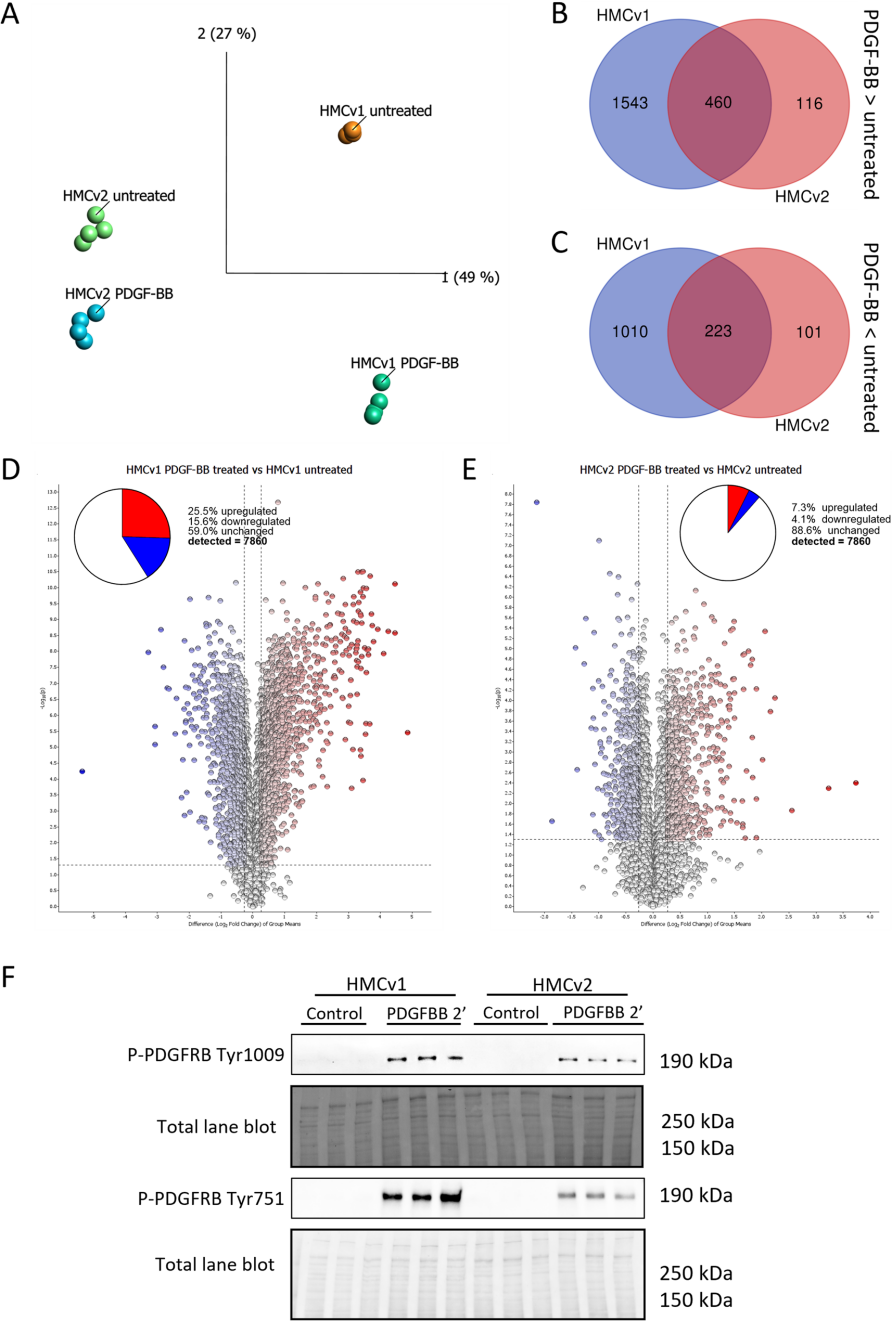
Proteomics, mesangial cell response to PDGF-BB stimulation. HMCv1 and HMCv2 were stimulated with 25ng/ml of PDGF-BB for 24 h and compared to their respective untreated controls (**A**). Dimensionality reduction via principal component analysis (PCA) showing separation between HMCv1 and 2 as well as separation between PDGF-BB treated cells and their respective controls. PDGF-BB treatment in HMCv1 and HMCv2 generated a partially overlapping regulation pattern at protein level, upregulated proteins (**B**) and downregulated proteins (**C**). Cutoffs for regulated proteins were ± 20% fold change and a *P* < 0.05. (**D**-**E**) Differences at protein expression level for both HMCv1 (**D**) and HMCv2 (**E**) are presented using volcano plots for the comparison between PDGF-BB treated and untreated cells. The dotted lines in the plots represent 0.05 p-value and ± 20% fold change thresholds. *n* = 4 experiments per group. Analysis was done using multigroup ANOVA with data filtering by standard deviation (S/Smax = 0.0003). *n* = 4 replicates per treatment, cell type. Phosphorylation of PDGFRB in HMCv1 and HMCv2 after 2 min treatment with PDGF-BB was investigated with western blot (**F**). Results are shown alongside the respective total protein blot (high molecular weights). The uncut blots are provided in the supplemental materials. *n* = 3 replicates per cell type and treatment

Principal component analysis (PCA) plots showed a clear separation between HMCv1 and HMCv2 both at baseline and after PDGF-BB stimulation. The extent of separation between untreated and treated HCMv1 was broader than HMCv2 untreated versus treated, revealing at a glance that HMCv1 response to PDGF-BB stimulation was stronger (Fig. 4A).

This was further shown (Fig. 4B-C) by the observation that HMCv1 had 2003 significantly upregulated and 1233 downregulated proteins when comparing PDGF-BB stimulated to unstimulated, while HMCv2 only had 576 upregulated and 324 downregulated proteins. Volcano plots showed that the distribution between upregulated and downregulated proteins after PDGF-BB stimulation had a similar pattern for both HMCv1 and 2, however, once again, PDGF-BB stimulation resulted in larger changes in the protein expression in HMCv1 than HMCv2 (Fig. 4D-E).

Finally, the protein levels of phosphorylated (ligand-binding activated) PDGFRB (respectively on Tyr1009 and Tyr751) were analyzed to confirm PDGF-BB stimulation of HMCs. Both MCs showed an extensive phosphorylation of PDGFRB already at 2 min of PDGF-BB treatment, with HMCv1 showing the highest phosphorylation extent (Fig. 4F).

### Pathway analysis of PDGF-BB stimulated mesangial cells

**Fig. 5:**
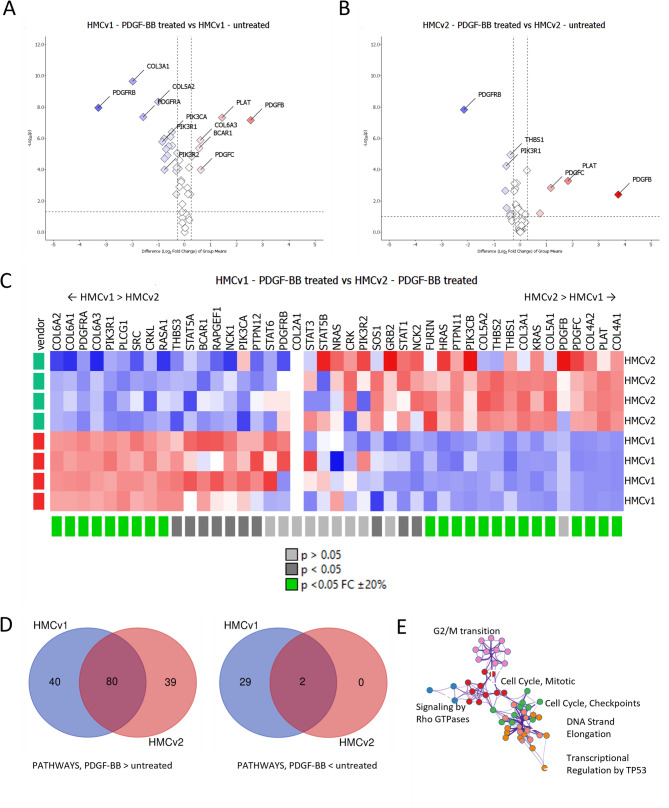
Proteomics pathway analysis after PDGF-BB stimulation. Volcano plots showing the regulated proteins in the Reactome PDGF pathway (R-HSA-186797). The dotted lines represent 0.05 p-value and ± 20% fold change thresholds (**A**-**B**). Heat map showing the differential regulation of the PDGF signaling pathway in HMCv1 versus HMCv2 (**C**). Fold change range was scaled from − 2 to + 2. P values and fold changes thresholds are reported in the legend. Venn diagrams showing the results from the total pathway analysis for the PDGF-BB treated versus untreated comparison, respectively for HMCv1 and 2 (**D**). Network showing the most regulated pathways for both HMCv1 and 2 after PDGF-BB stimulation (**E**)

To get further insight into the effect of PDGF-BB stimulation on the cells, the PDGF signaling pathway was further explored. 43 out of the total 58 proteins belonging to the Reactome PDGF signaling pathway (R-HAS-186707) were found for both HMCv1 and 2. The number of significantly regulated proteins (*P* < 0.05 and fold change ± 20%) was 20 (6 up-, 14 downregulated) for HMCv1 and 8 (3 up-, 5 downregulated) for HMCv2, with both MCs activating a negative feedback loop (downregulation of PDGFRB). Both MCs displayed an upregulation of PDGF-C and tissue-type plasminogen activator (PLAT), an activator of latent PDGF-CC [25]. PDGF-B levels were high in both HMCv1 and 2, but there is no way to distinguish if this is PDGF-B produced by the cells or the PDGF-BB used to stimulate the cells. (Fig. 5A-B). There were also differences in how the proteins were regulated by the cells, as seen in the heat map in Fig. 5C. When comparing HMCv1 to HMCv2 it is clear that PDGF-BB stimulation activates the regulation of more proteins in HMCv1 than HMCv2, once more pointing towards HMCv1 being more reactive than HMCv2.

Pathway analysis of the proteomics datasets revealed that PDGF-BB stimulation led to upregulation of 120 pathways for HMCv1 and 119 for HMCv2. 31 pathways were significantly downregulated in HMCv1 and only 2 in HMCv2 (Fig. 5D). Out of the 25 top upregulated pathways for both HMCv1 and HMCv2, the majority were pathways involved in mitosis (Tables 2 and 3), which fits with PDGF-BB stimulation leading to increased proliferation of the cells. HMCv2 only had two pathways with significant downregulation, namely “smooth muscle contraction” and “molecules associated with elastic fibers” (Tables 2 and 3). Both these pathways were also found among the downregulated pathways for the HMCv1. Other significantly downregulated pathways in HMCv1 were mainly connected to matrix and cytoskeleton regulation (Table 2). Cluster analysis of the most regulated pathways in the comparison between HMCv1 and HMCv2 cells showed that pathways downstream of PDGF signaling are activated in both, with mitosis upregulation being unsurprisingly the main activated pathway (Fig. 5E).

**Table 2:** Top 25 most upregulated and downregulated pathways. PDGF-BB treated vs. Untreated. HMCv1

Pathway. upregulated	proteins found	total proteins	ratio	*p* value	q value	minusLOG *p* value	minusLOG q value
Mitotic Prometaphase	92	211	0.014	1.11E-16	7.03E-14	15.95	13.15
Cell Cycle. Mitotic	211	595	0.039	1.11E-16	7.03E-14	15.95	13.15
Cell Cycle	248	733	0.048	1.11E-16	7.03E-14	15.95	13.15
Cell Cycle Checkpoints	98	279	0.018	1.74E-14	8.28E-12	13.76	11.08
rRNA modification in the nucleus and cytosol	43	71	0.005	3.39E-14	1.29E-11	13.47	10.89
M Phase	126	415	0.027	1.01E-13	3.18E-11	13.00	10.50
Mitotic G1 phase and G1/S transition	67	174	0.011	5.36E-12	1.31E-09	11.27	8.88
G1/S Transition	61	150	0.010	5.53E-12	1.31E-09	11.26	8.88
Mitotic Spindle Checkpoint	50	111	0.007	1.41E-11	2.97E-09	10.85	8.53
S Phase	67	179	0.012	1.77E-11	3.36E-09	10.75	8.47
DNA strand elongation	27	38	0.003	6.03E-11	9.53E-09	10.22	8.02
Amplification of signal from the kinetochores	44	94	0.006	7.10E-11	9.58E-09	10.15	8.02
Amplificationof signal from unattachedkinetochores via a MAD2inhibitory signal	44	94	0.006	7.10E-11	9.58E-09	10.15	8.02
Major pathway of rRNA processing in the nucleolus and cytosol	66	189	0.012	4.06E-10	5.12E-08	9.39	7.29
rRNA processing in the nucleus and cytosol	70	207	0.014	4.56E-10	5.38E-08	9.34	7.27
Activation of gene expression by SREBF (SREBP)	35	71	0.005	1.64E-09	1.82E-07	8.79	6.74
Activation of the pre-replicative complex	24	36	0.002	2.30E-09	2.30E-07	8.64	6.64
Resolution of Sister Chromatid Cohesion	51	134	0.009	2.56E-09	2.43E-07	8.59	6.61
Recruitment of mitotic centrosome proteins and complexes	37	81	0.005	4.15E-09	3.74E-07	8.38	6.43
AURKA Activation by TPX2	35	74	0.005	4.58E-09	3.94E-07	8.34	6.40
G2/M Transition	68	212	0.014	6.26E-09	5.14E-07	8.20	6.29
Centrosome maturation	37	83	0.005	7.74E-09	6.11E-07	8.11	6.21
Mitotic G2-G2/M phases	68	214	0.014	8.95E-09	6.80E-07	8.05	6.17
rRNA processing	75	247	0.016	1.01E-08	7.35E-07	8.00	6.13
Synthesis of DNA	49	132	0.009	1.12E-08	7.48E-07	7.95	6.13

Pathways. downregulated	Proteins found	Total proteins	Ratio	*p* value	q value	minusLOG *p* value	minusLOG q value
Extracellular matrix organization	69	328	0.022	1.97E-09	2.26E-06	8.71	5.65
Non-integrin membrane-ECM interactions	25	61	0.004	2.56E-09	2.26E-06	8.59	5.65
Integrin cell surface interactions	29	86	0.006	1.04E-08	6.10E-06	7.98	5.21
ECM proteoglycans	25	79	0.005	3.32E-07	1.46E-04	6.48	3.84
Syndecan interactions	14	29	0.002	1.22E-06	4.30E-04	5.91	3.37
RHO GTPase cycle	75	460	0.030	7.01E-06	2.06E-03	5.15	2.69
Axon guidance	89	585	0.039	1.38E-05	3.26E-03	4.86	2.49
Nervous system development	93	621	0.041	1.61E-05	3.26E-03	4.79	2.49
Collagen degradation	20	69	0.005	1.66E-05	3.26E-03	4.78	2.49
Regulation of Insulin-like Growth Factor (IGF) transport and uptake by IGFBPs	29	127	0.008	2.19E-05	3.54E-03	4.66	2.45
Cell-Cell communication	30	134	0.009	2.28E-05	3.54E-03	4.64	2.45
Response to elevated platelet cytosolic Ca2+	32	148	0.010	2.41E-05	3.54E-03	4.62	2.45
Collagen chain trimerization	15	44	0.003	3.09E-05	4.17E-03	4.51	2.38
Nephrin family interactions	11	25	0.002	3.77E-05	4.64E-03	4.42	2.33
MET promotes cell motility	15	45	0.003	3.97E-05	4.64E-03	4.40	2.33
Neutrophil degranulation	74	478	0.032	4.25E-05	4.67E-03	4.37	2.33
Post-translational protein phosphorylation	25	109	0.007	7.28E-05	7.25E-03	4.14	2.14
Molecules associated with elastic fibres	13	37	0.002	7.57E-05	7.25E-03	4.12	2.14
Platelet activation. signaling and aggregation	50	293	0.019	7.88E-05	7.25E-03	4.10	2.14
Smooth Muscle Contraction	17	61	0.004	1.10E-04	9.34E-03	3.96	2.03
Signaling by Rho GTPases	99	708	0.047	1.11E-04	9.34E-03	3.95	2.03
Degradation of the extracellular matrix	30	148	0.010	1.30E-04	9.97E-03	3.89	2.00
Platelet degranulation	29	141	0.009	1.31E-04	9.97E-03	3.88	2.00
Elastic fibre formation	14	45	0.003	1.44E-04	1.05E-02	3.84	1.98
Signaling by Rho GTPases. Miro GTPases and RHOBTB3	100	724	0.048	1.58E-04	1.11E-02	3.80	1.95

**Table 3:** Top 25 most upregulated and downregulated pathways. PDGF-BB treated vs. Untreated. HMCv2

Pathways. upregulated	proteins found	total proteins	ratio	*p* value	q value	minusLOG *p* value	minusLOG q value
Mitotic G1 phase and G1/S transition	46	174	0.011	1.11E-16	2.48E-14	15.95	13.61
Cell Cycle. Mitotic	113	595	0.039	1.11E-16	2.48E-14	15.95	13.61
Mitotic Prometaphase	44	211	0.014	1.11E-16	2.48E-14	15.95	13.61
Cell Cycle	127	733	0.048	1.11E-16	2.48E-14	15.95	13.61
G1/S Transition	40	150	0.010	1.11E-16	2.48E-14	15.95	13.61
Cell Cycle Checkpoints	52	279	0.018	1.11E-16	2.48E-14	15.95	13.61
M Phase	58	415	0.027	3.00E-14	5.67E-12	13.52	11.25
Resolution of Sister Chromatid Cohesion	32	134	0.009	3.40E-14	5.67E-12	13.47	11.25
Amplification of signal from the kinetochores	27	94	0.006	5.10E-14	6.83E-12	13.29	11.17
Amplificationof signal from unattachedkinetochores via a MAD2inhibitory signal	27	94	0.006	5.10E-14	6.83E-12	13.29	11.17
Mitotic Spindle Checkpoint	29	111	0.007	6.13E-14	7.48E-12	13.21	11.13
EML4 and NUDC in mitotic spindle formation	29	121	0.008	4.98E-13	5.52E-11	12.30	10.26
G1/S-Specific Transcription	18	43	0.003	2.06E-12	2.13E-10	11.69	9.67
S Phase	32	179	0.012	5.76E-11	5.47E-09	10.24	8.26
RHO GTPases Activate Formins	29	149	0.010	6.66E-11	5.93E-09	10.18	8.23
Mitotic Metaphase and Anaphase	38	250	0.016	9.32E-11	7.74E-09	10.03	8.11
Separation of Sister Chromatids	33	195	0.013	1.15E-10	8.99E-09	9.94	8.05
Activation of gene expression by SREBF (SREBP)	20	71	0.005	1.33E-10	9.86E-09	9.88	8.01
G2/M Transition	34	212	0.014	2.38E-10	1.67E-08	9.62	7.78
Mitotic G2-G2/M phases	34	214	0.014	3.02E-10	1.92E-08	9.52	7.72
Mitotic Anaphase	37	249	0.016	3.04E-10	1.92E-08	9.52	7.72
DNA strand elongation	14	38	0.003	2.87E-09	1.67E-07	8.54	6.78
Regulation of cholesterol biosynthesis by SREBP (SREBF)	20	87	0.006	4.06E-09	2.23E-07	8.39	6.65
Polo-like kinase mediated events	11	23	0.002	1.05E-08	5.56E-07	7.98	6.25
G0 and Early G1	13	38	0.003	2.47E-08	1.26E-06	7.61	5.90

Pathways. downregulated	Proteins found	Total proteins	Ratio	*p* value	q value	minusLOG *p* value	minusLOG q value
Smooth Muscle Contraction	10	61	0.004	3.92E-06	4.00E-03	5.41	2.40
Molecules associated with elastic fibres	7	37	0.002	4.59E-05	2.34E-02	4.34	1.63
*Elastic fibre formation*	7	45	0.003	1.53E-04	5.20E-02	3.82	1.28
*Extracellular matrix organization*	20	328	0.022	2.48E-04	5.60E-02	3.61	1.25
*Muscle contraction*	16	232	0.015	2.74E-04	5.60E-02	3.56	1.25
*Semaphorin interactions*	8	71	0.005	4.55E-04	7.74E-02	3.34	1.11
*Signaling by TGFB family members*	11	136	0.009	6.88E-04	9.98E-02	3.16	1.00
*Signaling by TGF-beta Receptor Complex*	9	107	0.007	1.59E-03	1.91E-01	2.80	0.72
*TGF-beta receptor signaling activates SMADs*	6	50	0.003	1.69E-03	1.91E-01	2.77	0.72
*Regulation of commissural axon pathfinding by SLIT and ROBO*	3	12	0.001	3.46E-03	2.96E-01	2.46	0.53
*Sema4D induced cell migration and growth-cone collapse*	4	25	0.002	3.68E-03	2.96E-01	2.43	0.53
*Oncogene Induced Senescence*	5	42	0.003	4.21E-03	2.96E-01	2.38	0.53
*Non-integrin membrane-ECM interactions*	6	61	0.004	4.46E-03	2.96E-01	2.35	0.53
*Regulation of cortical dendrite branching*	2	4	0.000	4.57E-03	2.96E-01	2.34	0.53
*FOXO-mediated transcription of cell cycle genes*	4	27	0.002	4.82E-03	2.96E-01	2.32	0.53
*HS-GAG degradation*	5	44	0.003	5.10E-03	2.96E-01	2.29	0.53
*Intracellular signaling by second messengers*	18	368	0.024	5.30E-03	2.96E-01	2.28	0.53
*CD28 dependent PI3K/Akt signaling*	4	28	0.002	5.47E-03	2.96E-01	2.26	0.53
*Integrin cell surface interactions*	7	86	0.006	6.08E-03	2.96E-01	2.22	0.53
*Glycosphingolipid metabolism*	7	86	0.006	6.08E-03	2.96E-01	2.22	0.53
*Syndecan interactions*	4	29	0.002	6.18E-03	2.96E-01	2.21	0.53
*Sema4D in semaphorin signaling*	4	31	0.002	7.76E-03	3.57E-01	2.11	0.45
*Surfactant metabolism*	5	52	0.003	1.00E-02	4.42E-01	2.00	0.35
*RUNX1 regulates genes involved in megakaryocyte differentiation and platelet function*	6	76	0.005	1.23E-02	4.61E-01	1.91	0.34
*Post-transcriptional silencing by small RNAs*	2	7	0.000	1.33E-02	4.61E-01	1.88	0.34

Pathways in italic: FDR > 0.05

### Inflammatory response to IL-1β treatment

**Fig. 6:**
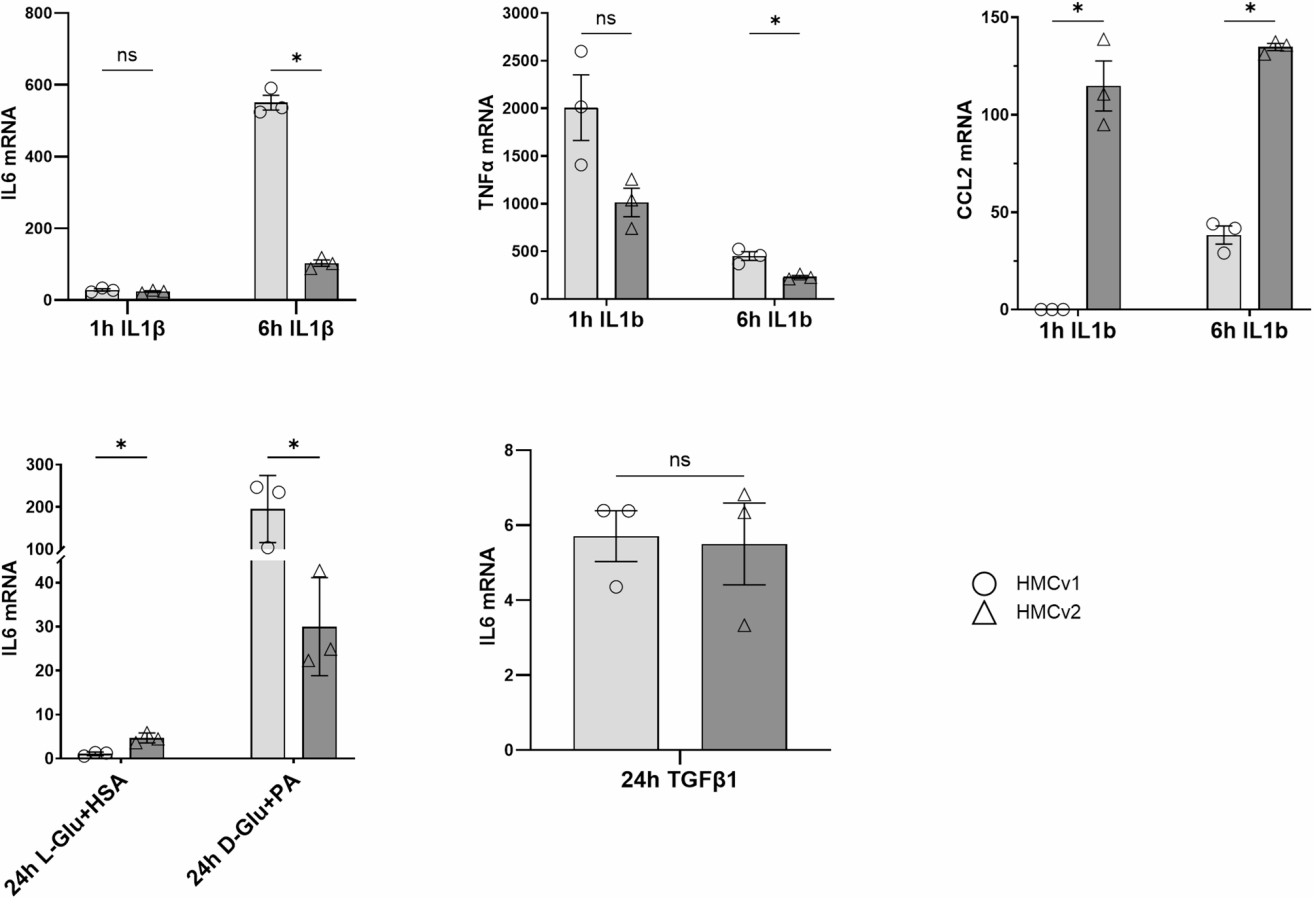
Inflammatory stimulus with IL-1β, diabetic milieu and TGFβ. HMCv1 and HMCv2 responded differentially to a 6 h IL-1β and 24 h diabetic milieu stimulation. Expression of IL6 (**A**), TNFα (**B**) and MCP1/CCL2 (**C**) was studied at gene level comparing the levels recorded at 1 h and 6 h of stimulation with IL-1β. IL-6 expression after 24 h of diabetic milieu (**D**) and TGFβ1 (**E**). *n* = 3 replicates per time point, condition and cell type. Error bars represent SEM, * *P* < 0.05, *** *P* < 0.001, t-test were used for the comparisons

To understand how MCs respond to inflammation, cells were treated with IL-1β for 1 h and 6 h. The gene expressions of IL6 (interleukin 6), TNFα (tumor necrosis factor alpha) and MCP1/CCL2 (chemokine C-C motif ligand 2 or monocyte chemoattractant protein-1) were compared to untreated controls at the same time point (Fig. 6). The gene expression of IL6 increased after 1 h with no significant difference between HMCv1 and 2. After 6 h the gene expression had risen even more with the highest upregulation found in HMCv1 (*P* < 0.001). For TNFα, the expression for both HMCv1 and 2 was highest at the 1 h time point with no significant difference between the MCs. At 6 h TNFα levels were decreased, with HMCv1 showing a higher expression (*P* < 0.05). For MCP1/CCL2 the expression at 1 h was almost at the limit of detection for HMCv1 but already high in HMCv2 (*P* < 0.05), at 6 h HMCv1 had started to increase the expression while the expression for HMCv2 was slightly increased and still significantly higher than HMCv1 (*P* < 0.001).

### Response to diabetic milieu and TGFβ1 treatment

To understand how MCs respond to diabetic milieu, cells were treated with either high glucose in combination with palmitate or L-glucose and HSA (osmotic control) or TGFβ1 and compared to untreated cells. HMCv1 had significantly higher expression of IL-6 after treatment for 24 h (*P* < 0.05) than HMCv2. For TGFβ treated cells the IL-6 expression increased but there was no difference between the two cell sources.

### Comparison of HMCv1 and HMCv2 with a third HMC source

**Fig. 7:**
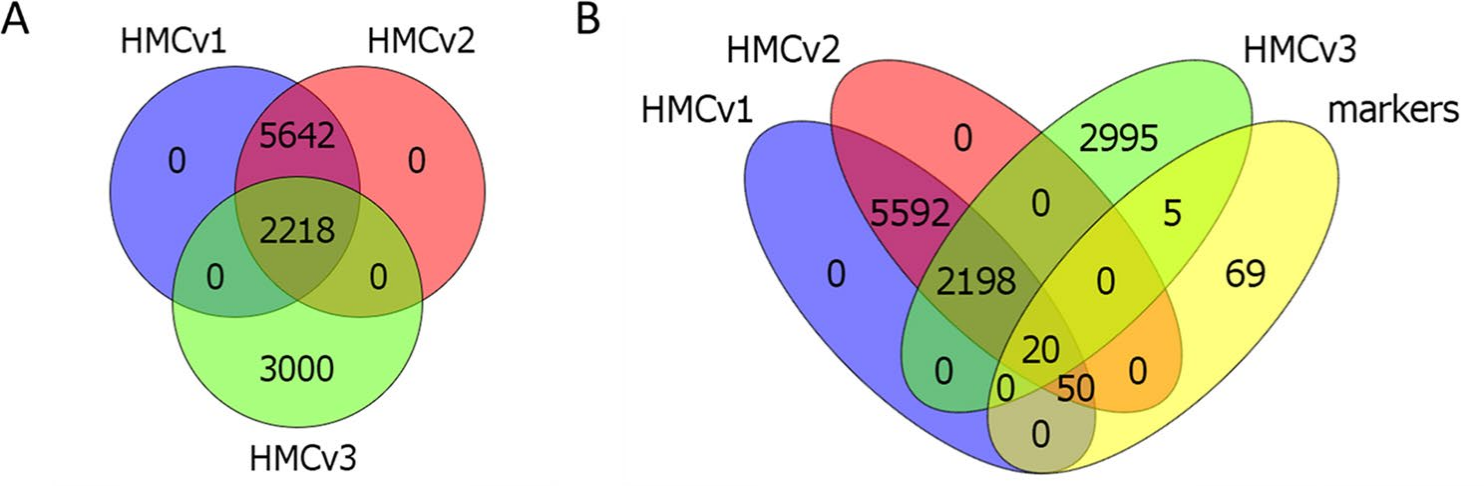
Protein expressions in HMCs from three different vendors. The list of quantified proteins from HMCv1 and HMCv2 were compared to previously published data of human mesangial cells from a third source, HMCv3 (**A**). The list of identified proteins in untreated cells was compared. 2218 common proteins were identified in all three mesangial cells (**B**). HMCv3 expressed only 25 mesangial markers (5 unique), whereas HMCv1 and HMCv2 both expressed 70

The list of identified proteins in unstimulated HMCv1 and HMCv2 was compared to a previously published proteomic dataset of unstimulated HMCs from another source (HMCv3) [10]. In total, 5218 proteins were quantified in HMCv3 and of those, only 2218 proteins overlapped with those found in both HMCv1 and 2 (Fig. 7A). Moreover, only 25 mesangial markers were identified in the HMCv3, among those, 20 were in common with HMCv1 and 2 (Fig. 7B). This data supports once again the notion that there are broad differences between primary MCs from different sources/donors.

## Discussion

One of the challenges of working with human primary cells is that the cells can broadly differ between donors and this discrepancy can heavily influence the outcome of experiments. To illustrate how much the outcome is dependent on the source of the cells, we compared two commercially available primary HMCs from two different donors/vendors. First, cellular characteristics such as morphology and ability to contract and proliferate were characterized, and then the ability of the MCs to respond to inflammation (IL-1β), diabetic milieu, and proliferation (PDGF-BB) stimulation were investigated, with special interest in the latter, since it is a common mesangial pathological condition in many glomerulopathies.

Overall, HMCv1 was characterized by a more active and responsive phenotype than HMCv2, although HMCv2 had a more mesangial-like morphological appearance. Cells from both sources expressed the mesangial markers normally used for characterization (PDGFRA, PDGRB, ACTA2) and responded to PDGF-BB stimulation with increased proliferation. Both MCs contracted in response to angiotensin II, although only HMCv1 contraction was statistically significant. IL-1β stimulation showed a different response, with HMCv1 more active in propagating and escalating inflammation (via IL6 and TNFα), and HMCv2 more able to attract monocytes via CCL2/MCP1 expression.

HMCs are versatile cells, with smooth muscle, macrophage and fibroblast-like properties. They do not express any specific cell marker and are therefore more difficult to characterize than the other cell types of the glomerulus. An RNAseq single cell study performed by He et al. suggested that “true” MCs should express high levels of 4 principal markers: PDGFRA, PDGFRB, GATA3 and CNN1 [13]. Both HMCv1 and 2 expressed all these markers at gene and protein level, but GATA3 was not detected in the mass spectrometry experiments and had only a low expression at the RNA level. HMCv1 had a higher expression of PDGFRA and B than HMCv2 both at RNA level and protein level as confirmed by qPCR, western blot and mass spectrometry. Both HMCv1 and 2 expressed smooth muscle actin (ACTA2), though the expression of smooth muscle actin in MCs is still debated. It is clear that MCs in culture express it, but in vivo it is considered to be expressed only in activated/de-differentiated MCs in disease state and been described as a myofibroblast [26]. When culturing cells there is always a risk of losing some of the physiological features of their in vivo phenotypes and that the cells de-differentiate. The expression of smooth muscle actin in cultured MCs as well as the loss of expression of GATA3 could be due to this process.

Furthermore, we have observed the presence of KRT19 (keratin 19) in both HMCv1 and HMCv2 (at both gene level and protein level, data not shown). KRT19 should be a marker of parietal epithelial glomerular cells. In the mass spectrometry data, we also found expression of the glomerular endothelial marker coagulation factor VIII (F8), but the mass spectrometry coverage was very low and another endothelial marker, PECAM (CD31), was not detected. One podocyte marker was also found, SYNPO, while nephrin and podocin were absent. This is in accord with recent single cell data showing expression of SYNPO in the mesangium [20, 27].

These findings indicate that there may be some de-differentiation of the HMCs at play, and/or the presence of a limited number of other glomerular cells in the mesangial culture.

Using previously published datasets we established a list of 144 genes/proteins suggested to be expressed by MCs. The collection was used as a list of mesangial markers throughout the proteomics analysis. Among them, we found that both HMCv1 and HMCv2 expressed 70 of these markers. When comparing these results to a previously published proteomics data set [10] of human primary MCs from a third source, we found only 25 mesangial markers (70 in HMCv1 and 2). Only 5 markers were unique and 20 found in HMCv1 and 2 as well. This discrepancy could partly be due to technical differences in the mass spectrometry runs (the HMCv3 dataset was produced in 2017, thus methodological and technical advances are to be expected) and different culture conditions, but this clearly shows that primary MCs can differ between origins of the cells.

Another way to characterize MCs is to investigate their response to angiotensin II and PDGF-BB. MCs have been shown to contract in response to angiotensin II both in vitro [24, 28] and in vivo in rats [29]. HMCv1 did significantly contract when stimulated with angiotensin II, while HMCv2 contraction did not reach significant levels.

Stimulation with PDGF-BB revealed both similarities and differences between HMCv1 and HMCv2. PDGF-BB is the major growth factor when it comes to mesangial proliferation and MCs both have an autocrine and paracrine response to PDGF [30–33]. Both HMCv1 and 2 respond to PDGF-BB stimulation with significantly increased proliferation rate. But when examining the response to PDGF-BB stimulation in detail via mass spectrometry, HMCv1 showed a higher number of regulated proteins than HMCv2. In particular, HMCv1 exhibited more regulated proteins belonging to the PDGF signaling pathway. Nonetheless, both cells upregulated PDGF-C and plasminogen activator tissue type (PLAT) after stimulation, as well as downregulated PDGFRB, in a possible negative feedback mechanism. Moreover, HMCv1 had an increase in other activated pathways after stimulation when compared to HMCv2, though overall the regulation pattern was similar. Several pathways involved in different aspects of proliferation were represented in both cases in the list of significantly up-regulated pathways. Concerning the downregulated pathways, we observed only 2 in HMCv2, while HMCv1 had 31, probably due to the general lesser extent of regulation of HMCv2. The downregulated pathways were mainly associated with extracellular matrix dynamics. The lower response to PDGF-BB stimulation in HMCv2 could be a reflection of the lower expression of the PDGF receptors in these cells. Both the stimulation with PDGF-BB and angiotensin II pointed towards a lower response capacity of HMCv2 in respect to HMCv1.

Phosphorylation of PDGFRB was used to validate the binding of the ligand PDGFB to the receptor and that the activation of the receptor by autophosphorylation has taken place [34]. The levels of phospho-Tyr751 and 1009 after 2 min of stimulation with PDGF-BB were elevated in both HMCv1 and HMCv2, with HMCv1 showing the higher extent of phosphorylation. Tyr1009 phosphorylation is known to generate a binding site for phospholipase C gamma [35], while Tyr751 phosphorylation is related to phosphatidyl-inositol 3 kinase [36, 37]. Both phosphorylation sites relate to the proliferation stimulus mediated by PDGF signaling [38].

Stimulation with inflammatory cytokine IL-1β was performed to highlight differences in the inflammatory response in between HMCv1 and HMCv2. Inflammation is of special importance in pathologies such as diabetic kidney disease, hence the extent of response to IL-1β, diabetic milieu and TGFβ1 stimulation was deemed interesting to explore [39–41]. At 1 h of IL-1β stimulation, HMCv1 expressed more IL6 and TNFα than HMCv2. IL6 expression increased at 6 h (20-fold for HMCv1, 4-fold for HMCv2), while TNFα expression decreased with a similar pattern in both MCs. Concerning the expression of MCP1/CCL2, at 1 h the most responsive cells were HMCv2. At 6 h of stimulation, there was only a slight further increase for HMCv2 (ca. + 20%), while HMCv1 started the production of MCP1/CCL2 (40-fold increase, but still 3 times lower than HMCv2). We deduce that both cell types strongly react to IL-1β stimulation, but in a different way: HMCv2 seems to activate monocytes chemoattraction (via CCL2/MCP1) and HMCv1 is more prone to escalate inflammation (via IL6 and TNFα). When cells were subjected to diabetic milieu the IL-6 expression increased for both HMCv1 and 2, but was significantly higher for HMCv1 (around 7 times higher), similar to findings for IL-1β stimulation. TGFβ1 stimulation also increased IL-6 expression (6-fold) but was similar for both cell sources.

An issue to keep in mind when working with cultured MCs in vitro is that it is impossible to replicate all the features of the in vivo environment, where MCs are in close contact with the glomerular endothelial cells and only separated from the podocytes by the basement membrane. Crosstalk between the cells of the glomerulus has been shown to be of importance both in healthy and diseased states. MCs have been shown to communicate both with podocytes, glomerular endothelial cells, as well as infiltrating immune cells [4]. For example, during development, PDGF-BB released from the glomerular endothelium is needed for the mesangium to form [42, 43]. When culturing any of the glomerular cells in single culture, it is important to acknowledge that this crosstalk will be lost and might influence their phenotype and moreover the activation and response of the cultured cells to cytokines and other stressors. In addition, potential systemic factors reaching the cells though the blood as well as the shear stress induced by the blood flow are completely missing in vitro.

The HMCV1 and HMCV2 used in this paper comes from two donors with different sexes, and there is a possibility that some of the differences found in this paper could be due to sex, which should be kept in mind when interpreting the data.

Even with all the differences and possible shortcomings shown in the choice of cells from different sources, primary MCs remain the best option for studying human glomerular MCs molecular mechanisms and pathophysiology. To date, there seems to be only one cell line of human MCs commercially available. It is a conditionally immortalized human mesangial cell line established by Sarrab et al. in 2011 expressing the markers ACTA2 and PDGFRB [44]. Furthermore, the lack of specific mesangial protein markers makes mesangial specific knockdown of proteins in mouse models (such as the podocin CRE for podocytes) practically impossible. Thus, researchers must rely on primary MCs for their experimental work for targeted gene knockdown or overexpression experiments.

Since the choice of primary MCs appears to be mandatory for MCs research, we advocate for a careful choice of an adequate source of MCs when starting an experimental pipeline. In light of this, we have provided insights into how different the response of two commercially available MCs to traditionally used treatments could be. Mesangial researchers are advised to tailor the choice of cultured MCs depending on the experimental pipeline of their research hypothesis.

## Conclusion

In conclusion, we found that there were similarities as well as differences between the primary human mesangial cells from two different available sources investigated in this paper. Both HMCv1 and HMCv2 behaved as MCs in that they expressed the major mesangial markers and responded to PDGF-BB stimulation. However, the HMCv1 cells were much more responsive to PDGF-BB and showed increased angiotensin II driven contractility compared to HMCv2. Response to inflammatory stimulation with IL-1β was also different between HMCv1 and HMCv2.

These differences could have a major impact on the outcome of experiments performed using these cells, and therefore we suggest that the cells’ response to different stimulants should be carefully considered before conducting experiments on primary HMCs. We also conclude that the proteomic dataset and the curated list of mesangial genes provided could be helpful for further investigation of the role of MCs in kidney disease.

## Supplementary Information

Below is the link to the electronic supplementary material.

Supplementary Material 1

Supplementary Material 2

Supplementary Material 3

Supplementary Material 4

Supplementary Material 5

Supplementary Material 6

Supplementary Material 7

Supplementary Material 8

Supplementary Material 9

Supplementary Material 10

Supplementary Material 11

Supplementary Material 12

Supplementary Material 13

Supplementary Material 14

Supplementary Material 15

## Data Availability

The mass spectrometry proteomics data have been deposited to the ProteomeXchange Consortium via the PRIDE (https://www.ebi.ac.uk/pride/) [45, 46] partner repository with the dataset identifier PXD060148.

## Abbreviations

AT II: Angiotensin II
CNN1: Calponin 1
CCL2: Chemokine C-C motif ligand 2
DKD: Diabetic kidney disease
FC: Fold change
GATA3: G-A-T-A containing sequence binding protein 3
HMCs: Human mesangial cells
IgAN: IgA nephropathy
IL-1β: Interleukin 1 bet
IL6: Interleukin 6
KRT19: Keratin 19
MCs: Mesangial cells
MCP1: Monocyte chemoattractant protein-1
NPHS1: Nephrin
PDGF: Platelet derived growth factor
PDGFRA: Platelet derived growth factor receptor alpha
PDGFRB: Platelet derived growth factor receptor alpha
NPHS2: Podocin
PCA: Principal component analysis
ACTA2: Smooth muscle actin
SYNPO: Synaptopodin
PLAT: Tissue-type plasminogen activator
TNFα: Tumor necrosis factor alpha
