# Supplementary material for: Comparative analysis of commercial human primary mesangial cell, implications for experimental design
Source: BMC Nephrol. 2025 Sep 29;26:539. doi: 10.1186/s12882-025-04444-1 (PMC12482395; doi:10.1186/s12882-025-04444-1)
Supplement: Supplementary file 1 — Supplementary Material 1 [file 12882_2025_4444_MOESM1_ESM.docx]

**Supplementary materials to:**

**Comparative analysis of commercial human primary mesangial cell, implications for experimental design.**

Alva Johansson, Gayathri Narasimhan, Katharina Keuenhof, Roberto Boi, Kerstin Ebefors

Institute of Neuroscience and Physiology, Sahlgrenska Academy, University of Gothenburg, Sweden

*Supplementary content:*

Supplementary method: Proteomics complete method

Supplementary figure 1. Full gel/blots from western blots for Figure 1C and 4F

Supplementary figure 2. Expression of markers for glomerular endothelial cells and podocytes in HMCv1 and HMCv2

Supplementary table 1. Human mesangial cells markers from recent literature

**Supplementary method**

*Proteomics complete method: Protein extraction, digestion, mass spectrometry and protein identification*

Protein concentration of cell lysates was determined using Pierce BCA Protein Assay Kit (Thermo Scientific) on a Benchmark Plus microplate reader (BIO-RAD). Aliquots of 40µg protein were reduced in 10 mM dithiothreitol at 56°C for 30 min, then alkylated with 20mM iodoacetamide at room temperature, and quenched with 10mM dithiothreitol for 10min.

Protein samples were added to washed hydrophobic and hydrophilic Sera-Mag™ SpeedBeads (Carboxylate-modified, Cytiva) in a bead to protein ratio of 10:1. The SP3-workflow was adapted from the protein and peptide clean-up for mass spectrometry protocol provided by the manufactorer. In short, proteins were precipitated on the beads by 100% ethanol, washed with 80% ethanol and dried at room temperature. For digestion, 50mM TEAB and 1.6µg LysC+trypsin (Promega) was added, and incubation took place overnight at 37°C while shaking. An additional portion of 0.8µg trypsin (Thermo Fisher Scientific) was added and digestion continued for four hours.

Peptides from the supernatant were labelled using TMTpro 18-plex isobaric mass tagging reagents (Thermo Fisher Scientific) according to the manufacturer´s instructions. All labelled samples were pooled into the different TMT-sets. Peptides were purified using HiPPR detergent removal kit and Pierce peptide desalting spin columns (both Thermo Fisher Scientific), according to the manufacturer´s instructions. The TMT-sets were fractionated by basic reversed-phase chromatography using a Dionex Ultimate 3000 UPLC system (Thermo Fisher Scientific). Peptide separations were performed using a reversed-phase XBridge BEH C18 column (3.5 μm, 2.1x250 mm, Waters Corporation) and a stepped gradient from 5% to 54% solvent B over 65 min followed by an increase to 80% B at a flow of 200 µL/min. Solvent A was 25mM ammonia and solvent B was 85% acetonitrile. The 96 primary fractions were combined into 30 final fractions which were evaporated and reconstituted in 3% acetonitrile, 0.1% trifluoroacetic acid for LC-MS3 analysis.

The fractions (around 1.2µg each) were analysed on an Orbitrap Eclipse Tribrid mass spectrometer equipped with the FAIMS Pro ion mobility system and interfaced with an Easy-nLC1200 liquid chromatography system (both Thermo Fisher Scientific). Peptides were trapped on an Acclaim Pepmap 100 C18 trap column (100 μm x 2 cm, particle size 5 μm, Thermo Fisher Scientific) and separated on an in-house packed analytical column (35 cm x 75 μm, particle size 3 μm, Reprosil-Pur C18, Dr. Maisch) using a stepped gradient from 4% to 80% acetonitrile in 0.2% formic acid over 75 min at a flow of 300 nL/min. FAIMS Pro was alternating between the compensation voltages (CV) of -50 and -70, and the same data-dependent settings were used at both CVs. The precursor ion mass spectra were acquired at a resolution of 120 000 and an m/z range of 375-1500. Using a cycle time of 1.5 seconds the most abundant precursors with charges 2–7 were isolated with an m/z window of 0.7 and fragmented by collision induced dissociation (CID) at 30%. Fragment spectra were recorded in the ion trap at Rapid scan rate. The ten most abundant MS2 fragment ions were isolated using multi-notch isolation for further MS3 fractionation. MS3 fractionation was performed using higher-energy collision dissociation (HCD) at 55% and the MS3 spectra were recorded in the Orbitrap at 50 000 resolution and an m/z range of 100–500.

Identification and relative quantification were performed using Proteome Discoverer version 2.4 (Thermo Fisher Scientific). The data was matched against Homo sapiens SwissProt database (20261 entries, May 2022). Database matching was performed using Sequest as a search engine with a precursor tolerance of 5 ppm and a fragment ion tolerance of 0.6 Da. Tryptic peptides were accepted with 0 missed cleavages; methionine oxidation was set as a variable modification and cysteine carbamidomethylation, TMTpro on lysine and peptide N-termini were set as fixed modifications. Percolator was used for PSM validation with a strict FDR threshold of 1%. For quantification TMT reporter ions were identified in the MS3 HCD spectra with 3 mmu mass tolerance and the TMT reporter intensity values for each sample were normalized on the total peptide amount. SPS Mass Match threshold was set to 65%, a Sequest XCorr threshold score of 1 was chosen. Only unique peptides were used for relative quantification and proteins were required to pass a protein FDR of 5%.

**Supplementary figure 1. Full gel/blots of western blots for Figure 1C**


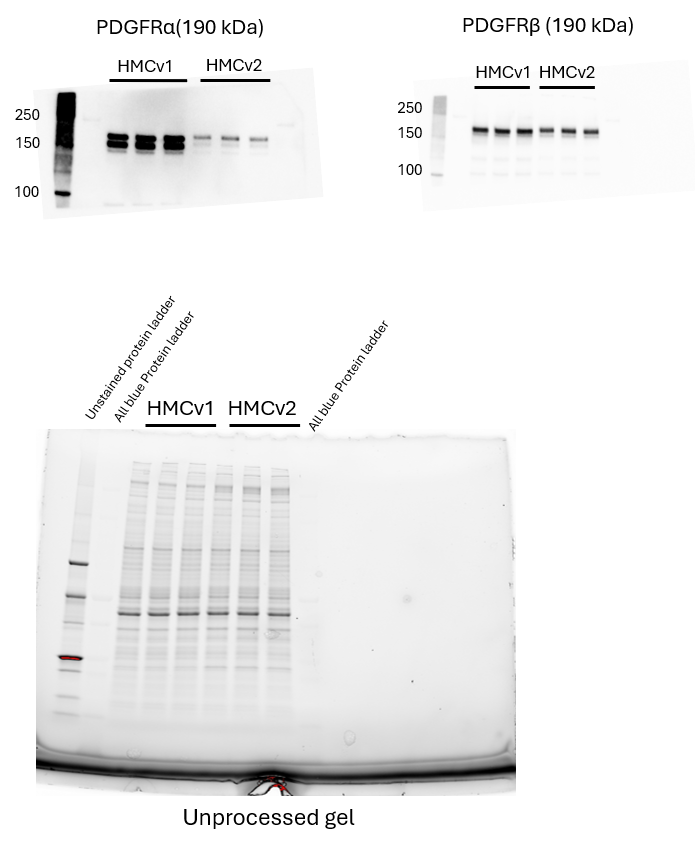
**
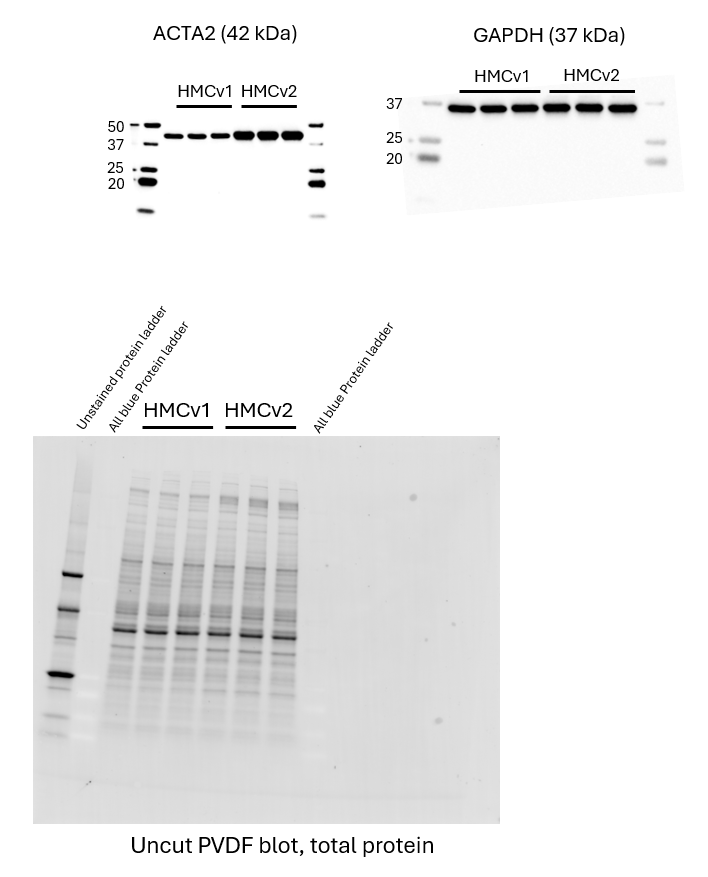
**

The same gel/blot was used for Figure 1C. The membrane was cut at 50 kDa. The upper part was used for PDGFRA, the membrane was then stripped and reprobed for PDGFRB. The lower part was used for ACTA2, the membrane was then stripped and reprobed for GAPDH.


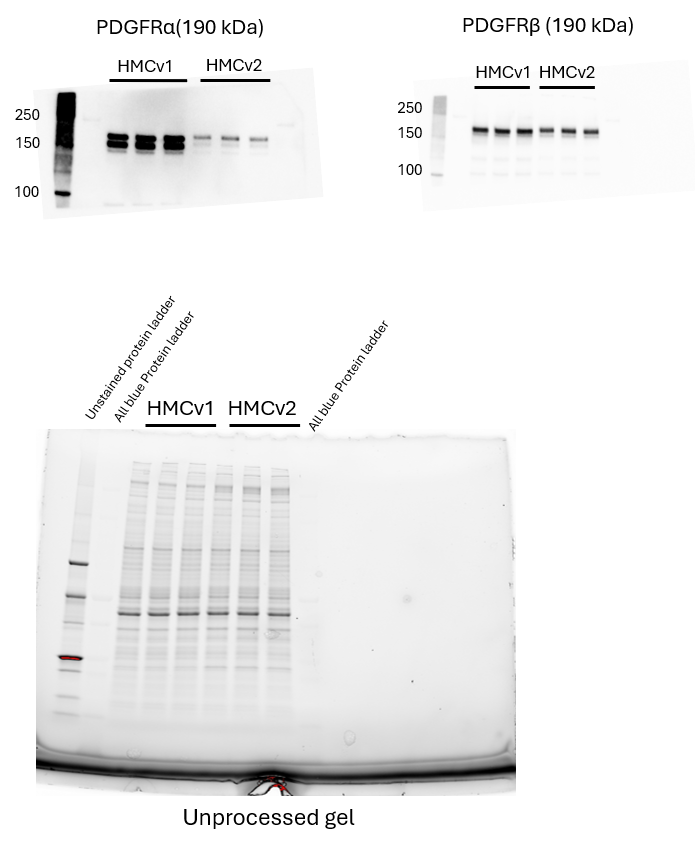


**
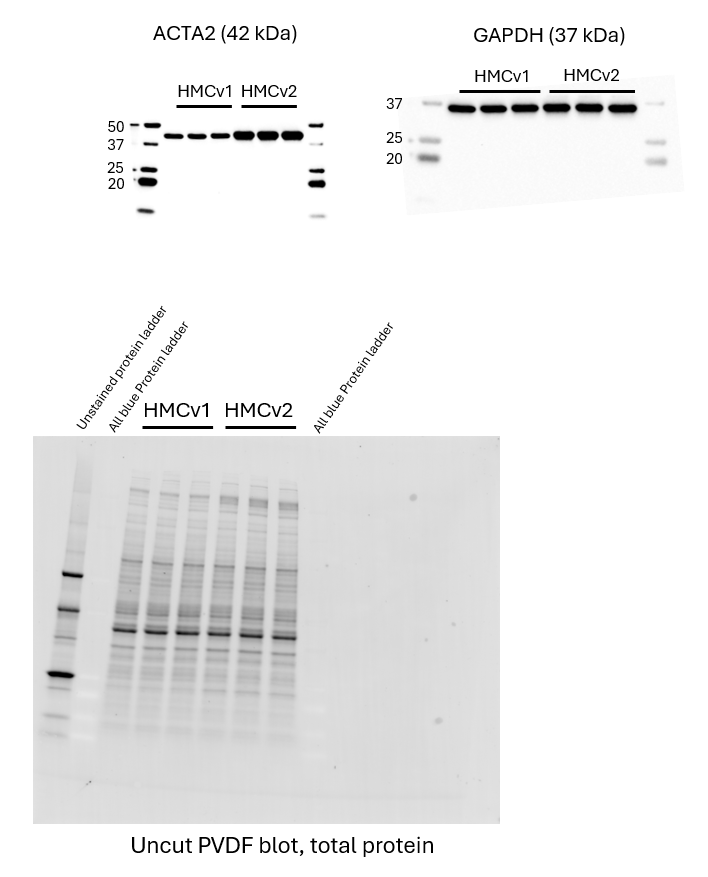
**

**Supplementary figure 1. Full gel/blots of western blots for Figure 4F**

Gel/blot 1 : PDGFRB phospho Y751


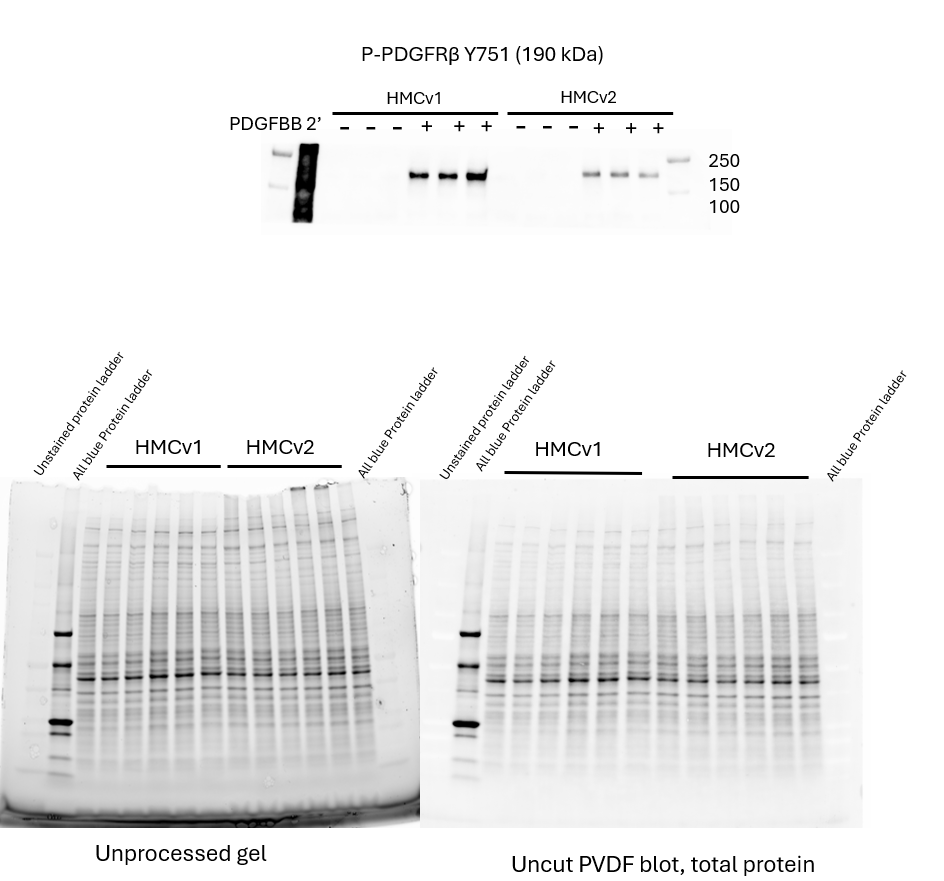


The PVDF membrane was cut at 100 kDa. The upper part was used for PDGFRB phospho Y751.

Gel/blot 1 : PDGFRB phospho Y1009

**
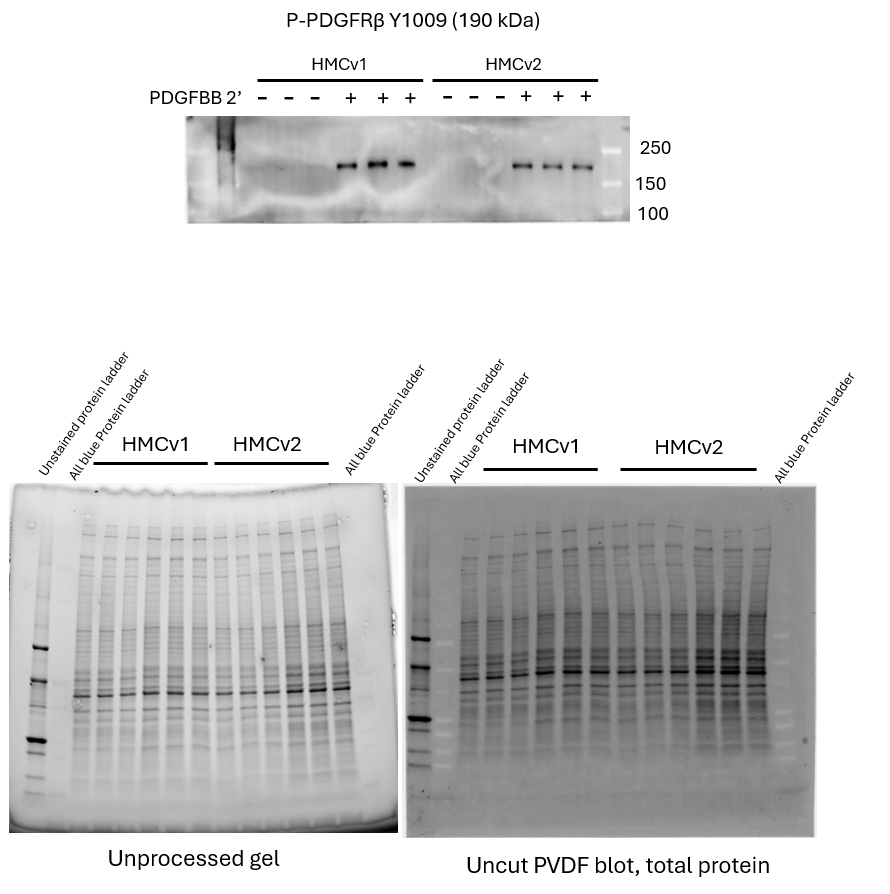
**

The PVDF membrane was cut at 100 kDa. The upper part was used for PDGFRB phospho Y1009.

**Supplementary figure 2. Expression of markers for glomerular endothelial cells and podocytes in HMCv1 and HMCv2.**

(A) Intensity of detected glomerular endothelial cells (HGEC) and podocytes (HPOD) markers. (B) Coverage and number of peptides detected in the mass spectrometry for selected proteins. **P<0.01, ***P<0.001, error bars represent SEM


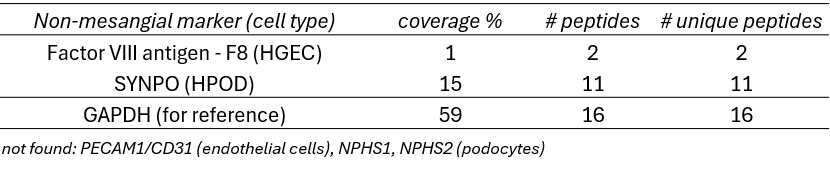


| **Supplementary table 1. Human mesangial cells markers from recent literature** | | | | | | | | | | | | | | | | |
| --- | --- | --- | --- | --- | --- | --- | --- | --- | --- | --- | --- | --- | --- | --- | --- | --- |
| *gene/protein* | *n obs* | *Hansen - Sci Adv. 2022 - ST5 - KPMP* | *Hansen - Sci Adv. 2022 - ST13 - KPMP* | *He - Nat commun 2021* | *Kuppe - Nature 2021* | *Lake - Nat commun 2019 - ST5* | *Lake - Nat commun 2019 - ST7* | *Lake - Nature 2023* | *Liu 2017 JASN HMC standard genes* | *Marshall - iScience 2022* | *Menon - JCI insight 2020* | *Muto - Nat Commun 2021* | *Wilson - Nat Commun 2022* | *Young - Science 2018 - ST2* | *Young - Science 2018 - ST4* | *Zhang - PNAS 2021* |
| PDGFRB | 11 | x | x |  | x |  | x |  | x | x |  | x | x | x | x | x |
| ACTA2 | 7 |  | x |  |  |  |  |  | x | x |  | x | x |  | x | x |
| AGTR1 | 7 | x | x |  |  | x | x |  | x |  |  | x | x |  |  |  |
| CALD1 | 7 | x |  |  | x |  | x |  | x | x |  | x | x |  |  |  |
| ITGA8 | 7 | x | x | x |  |  | x |  | x |  |  | x | x |  |  |  |
| EBF1 | 6 | x | x |  |  |  | x |  |  |  |  | x | x |  | x |  |
| PIEZO2 | 6 | x | x |  |  |  | x |  |  | x |  | x | x |  |  |  |
| ROBO1 | 6 | x | x |  |  |  | x |  |  | x |  | x | x |  |  |  |
| A2M | 5 | x |  |  | x |  | x |  |  |  |  | x | x |  |  |  |
| DAAM2 | 5 | x |  |  |  |  | x |  |  |  |  | x | x |  | x |  |
| GATA3 | 5 | x |  | x | x |  | x |  |  |  |  |  |  |  | x |  |
| PIP5K1B | 5 | x |  |  |  |  | x | x |  |  |  | x | x |  |  |  |
| COL12A1 | 4 | x |  |  |  |  | x |  |  |  |  | x | x |  |  |  |
| COL25A1 | 4 | x |  |  |  |  | x |  |  |  |  | x | x |  |  |  |
| DAPK2 | 4 | x |  |  |  |  | x |  |  |  |  | x | x |  |  |  |
| DLC1 | 4 | x |  |  |  |  | x |  |  |  |  | x | x |  |  |  |
| ENG | 4 | x |  |  |  |  | x |  |  |  |  | x | x |  |  |  |
| GJA5 | 4 | x |  |  |  |  |  |  |  |  |  | x | x |  | x |  |
| ITGA11 | 4 | x |  |  |  |  | x |  |  |  |  | x | x |  |  |  |
| KALRN | 4 | x |  |  |  |  | x |  |  |  |  | x | x |  |  |  |
| KCNE4 | 4 | x |  |  |  |  |  |  |  |  |  | x | x |  | x |  |
| MEF2C | 4 |  |  |  |  |  | x |  |  |  |  | x | x |  | x |  |
| MEIS1 | 4 | x |  |  |  |  | x |  |  |  |  | x | x |  |  |  |
| MEIS2 | 4 | x |  |  |  |  | x |  |  |  |  | x | x |  |  |  |
| MYH11 | 4 |  |  |  |  |  |  |  |  |  |  | x | x |  | x | x |
| NOTCH3 | 4 | x |  |  |  |  |  |  |  | x |  |  | x |  | x |  |
| NRGN | 4 | x |  |  | x |  |  |  |  |  |  | x | x |  |  |  |
| NTRK3 | 4 | x |  |  |  |  |  |  |  |  |  | x | x |  | x |  |
| PDE3A | 4 | x |  |  |  |  | x |  |  |  |  | x | x |  |  |  |
| POSTN | 4 | x | x |  |  | x | x |  |  |  |  |  |  |  |  |  |
| PRR16 | 4 | x |  |  |  |  | x |  |  |  |  | x | x |  |  |  |
| RBMS3 | 4 | x |  |  |  |  | x |  |  |  |  | x | x |  |  |  |
| SLCO3A1 | 4 | x |  |  |  |  | x |  |  |  |  | x | x |  |  |  |
| SLIT3 | 4 |  |  |  |  |  |  |  |  | x |  | x | x |  | x |  |
| TAGLN | 4 |  | x |  |  |  |  |  | x |  | x |  |  |  | x |  |
| TMTC1 | 4 | x |  |  |  |  | x |  |  |  |  | x | x |  |  |  |
| ROBO1 | 2 |  |  |  |  | x |  | x |  |  |  |  |  |  |  |  |
| ADCY3 | 3 | x |  |  |  |  |  |  |  |  |  | x | x |  |  |  |
| ADIRF | 3 |  |  |  | x |  |  |  |  |  |  | x | x |  |  |  |
| ANGPT2 | 3 |  |  |  |  |  |  |  | x |  |  | x |  |  | x |  |
| ANO3 | 3 | x |  |  |  |  |  |  |  |  |  | x | x |  |  |  |
| ATP10A | 3 | x |  |  |  |  |  |  |  |  |  | x | x |  |  |  |
| BGN | 3 | x |  |  | x |  |  |  |  |  |  |  |  |  | x |  |
| CARMN | 3 |  |  |  |  |  |  |  |  |  |  | x | x |  | x |  |
| CSPG4 | 3 |  |  |  |  |  |  |  |  |  |  | x | x |  | x |  |
| EPAS1 | 3 | x |  |  | x |  | x |  |  |  |  |  |  |  |  |  |
| EPB41L2 | 3 | x |  |  |  |  | x |  |  |  |  | x |  |  |  |  |
| ETS1 | 3 | x |  |  |  |  | x |  | x |  |  |  |  |  |  |  |
| FBXL7 | 3 |  |  |  |  |  | x |  |  |  |  | x | x |  |  |  |
| FILIP1 | 3 |  |  |  |  |  |  |  |  |  |  | x | x |  | x |  |
| FRZB | 3 | x |  |  | x |  |  |  |  |  |  |  |  |  | x |  |
| HEYL | 3 |  |  |  |  |  |  |  |  |  |  | x | x |  | x |  |
| HS3ST3A1 | 3 | x |  |  |  |  |  |  |  |  |  | x | x |  |  |  |
| IL18R1 | 3 | x | x |  |  |  | x |  |  |  |  |  |  |  |  |  |
| INPP4B | 3 | x |  |  |  |  | x |  |  |  |  |  |  |  | x |  |
| LHFP | 3 | x |  |  |  |  | x |  |  |  |  |  |  |  | x |  |
| LMOD1 | 3 |  |  |  |  |  |  |  |  |  |  | x | x |  | x |  |
| MCAM | 3 |  |  |  |  |  |  |  |  |  |  | x | x |  | x |  |
| MFGE8 | 3 |  |  |  |  |  |  |  |  |  |  | x | x |  | x |  |
| MGP | 3 |  |  |  | x |  | x |  |  | x |  |  |  |  |  |  |
| MICAL2 | 3 | x |  |  |  |  |  |  |  |  |  | x | x |  |  |  |
| MRVI1 | 3 |  |  |  |  |  |  |  |  |  |  | x | x |  | x |  |
| PDGFRA | 3 |  |  | x |  |  |  |  | x |  |  |  | x |  |  |  |
| PRKG1 | 3 | x |  |  |  |  | x |  |  |  |  |  | x |  |  |  |
| RAPGEF5 | 3 |  |  |  |  |  | x |  |  |  |  | x | x |  |  |  |
| RP11-627D16.1 | 2 | x |  |  |  |  | x |  |  |  |  |  |  |  |  |  |
| SAMD3 | 3 | x |  |  |  |  |  |  |  |  |  | x | x |  |  |  |
| SYNE1 | 3 | x |  |  | x |  | x |  |  |  |  |  |  |  |  |  |
| SYNPO2 | 3 | x |  |  |  |  |  |  |  |  |  | x | x |  |  |  |
| TIMP3 | 3 | x |  |  | x |  | x |  |  |  |  |  |  |  |  |  |
| TINAGL1 | 3 | x |  |  | x |  |  |  |  |  |  |  |  |  | x |  |
| TRPC4 | 3 | x |  |  |  |  |  |  |  |  |  | x | x |  |  |  |
| UNC5C | 3 | x |  |  |  |  | x |  |  |  |  |  | x |  |  |  |
| WFDC1 | 3 |  |  |  |  |  |  |  |  |  |  | x | x |  | x |  |
| ZEB2 | 3 | x | x |  |  |  | x |  |  |  |  |  |  |  |  |  |
| CACNA1C | 2 |  |  |  |  |  |  |  |  |  |  | x | x |  |  |  |
| CD34 | 2 | x |  |  |  |  |  |  | x |  |  |  |  |  |  |  |
| FN1 | 2 |  |  |  |  |  |  |  |  |  |  | x | x |  |  |  |
| NDUFA4L2 | 2 |  |  |  |  |  |  |  |  |  |  |  |  | x | x |  |
| PTN | 2 |  | x |  |  |  |  |  |  |  |  |  |  |  | x |  |
| RGS5 | 2 |  | x |  |  |  |  |  |  |  |  |  |  |  | x |  |
| S1PR3 | 2 | x |  |  |  |  |  |  |  |  |  |  |  |  | x |  |
| SEPT4 | 2 |  |  | x |  |  |  |  |  |  |  |  |  |  | x |  |
| VIM | 2 |  |  |  | x |  |  |  |  |  |  |  |  |  |  | x |
| EMCN | 2 | x |  |  |  |  | x |  |  |  |  |  |  |  |  |  |
| HPSE2 | 2 |  |  |  |  |  |  |  |  |  |  | x | x |  |  |  |
| IGFBP5 | 2 |  |  |  |  |  | x |  | x |  |  |  |  |  |  |  |
| ITGB1 | 2 |  |  |  | x |  |  |  | x |  |  |  |  |  |  |  |
| SPARCL1 | 2 |  |  |  |  |  |  |  |  |  |  | x | x |  |  |  |
| TPM2 | 2 |  |  |  | x |  |  |  |  |  |  |  |  |  | x |  |
| GAS2 | 2 | x |  |  |  |  | x |  |  |  |  |  |  |  |  |  |
| GRID2 | 2 |  |  |  |  |  |  |  |  |  |  | x | x |  |  |  |
| KCNQ5 | 2 |  |  |  |  |  |  |  |  |  |  | x | x |  |  |  |
| LAMA2 | 2 | x |  |  |  |  | x |  |  |  |  |  |  |  |  |  |
| TXNIP | 2 |  |  |  | x |  |  |  | x |  |  |  |  |  |  |  |
| EDNRA | 2 |  |  |  |  |  |  |  |  |  |  | x | x |  |  |  |
| IL1RAPL1 | 2 | x |  |  |  |  | x |  |  |  |  |  |  |  |  |  |
| KCNAB1 | 2 |  |  |  |  |  |  |  |  |  |  | x |  |  | x |  |
| MYL9 | 2 |  |  |  | x |  |  |  |  |  |  |  |  |  | x |  |
| THY1 | 2 |  |  |  |  |  |  |  | x |  |  |  |  |  | x |  |
| ADAMTS6 | 2 |  |  |  |  |  |  |  |  |  |  | x | x |  |  |  |
| TNS1 | 2 |  |  |  |  |  | x |  | x |  |  |  |  |  |  |  |
| DGKG | 2 |  |  |  |  |  |  |  |  |  |  | x | x |  |  |  |
| EDIL3 | 2 |  |  |  |  |  |  |  |  |  |  | x | x |  |  |  |
| SPARC | 2 | x |  |  |  |  | x |  |  |  |  |  |  |  |  |  |
| WISP1 | 2 | x |  |  |  |  |  |  |  |  |  | x |  |  |  |  |
| COL21A1 | 2 |  |  |  |  |  |  |  |  |  |  | x | x |  |  |  |
| HSPA1B | 2 | x |  |  |  |  | x |  |  |  |  |  |  |  |  |  |
| CPED1 | 2 |  |  |  |  |  |  |  |  |  |  | x | x |  |  |  |
| SPOCK1 | 2 | x |  |  |  |  | x |  |  |  |  |  |  |  |  |  |
| ELN | 2 |  |  |  |  |  |  |  |  |  |  | x | x |  |  |  |
| HSPA1A | 2 | x |  |  |  |  | x |  |  |  |  |  |  |  |  |  |
| LZTS1 | 2 |  |  |  |  |  |  |  |  |  |  |  | x |  | x |  |
| FHL5 | 2 |  |  |  |  |  |  |  |  |  |  | x |  |  | x |  |
| PHTF2 | 2 | x |  |  |  |  | x |  |  |  |  |  |  |  |  |  |
| TCF4 | 2 | x |  |  |  |  | x |  |  |  |  |  |  |  |  |  |
| PALLD | 2 |  |  |  |  |  | x |  |  |  |  |  |  |  | x |  |
| ADRA1A | 2 |  |  |  |  |  |  |  |  |  |  | x | x |  |  |  |
| C7 | 2 | x |  |  |  |  | x |  |  |  |  |  |  |  |  |  |
| LDB3 | 2 |  |  |  |  |  |  |  |  |  |  | x | x |  |  |  |
| FOSB | 2 | x |  |  |  |  | x |  |  |  |  |  |  |  |  |  |
| MYOCD | 2 |  |  |  |  |  |  |  |  |  |  | x | x |  |  |  |
| PCDH9 | 2 | x |  |  |  |  | x |  |  |  |  |  |  |  |  |  |
| ZFPM2-AS1 | 2 | x |  |  |  |  |  |  |  |  |  |  | x |  |  |  |
| MCF2 | 2 |  |  |  |  |  |  |  |  |  |  | x | x |  |  |  |
| PRKCA | 2 | x |  |  |  |  | x |  |  |  |  |  |  |  |  |  |
| TBX3 | 2 | x |  |  |  |  |  |  |  |  |  |  | x |  |  |  |
| ANO1 | 2 |  |  |  |  |  |  |  |  |  |  | x | x |  |  |  |
| DNAJB1 | 2 | x |  |  |  |  | x |  |  |  |  |  |  |  |  |  |
| ADGRF5 | 2 | x |  |  |  |  | x |  |  |  |  |  |  |  |  |  |
| PDZRN4 | 2 |  |  |  |  |  |  |  |  |  |  | x | x |  |  |  |
| ITGA7 | 2 |  |  |  |  |  |  |  |  |  |  | x | x |  |  |  |
| KCNB1 | 2 |  |  |  |  |  |  |  |  |  |  | x | x |  |  |  |
| MSRB3 | 2 |  |  |  |  |  |  |  |  |  |  | x | x |  |  |  |
| MDGA2 | 2 |  |  |  |  |  |  |  |  |  |  | x | x |  |  |  |
| TMEM131L | 2 |  |  |  |  |  |  |  |  |  |  | x | x |  |  |  |
| MYOM1 | 2 |  |  |  |  |  |  |  |  |  |  | x | x |  |  |  |
| SLC7A2 | 2 |  |  |  |  |  |  |  |  |  |  | x | x |  |  |  |
| JPH2 | 2 |  |  |  |  |  |  |  |  |  |  | x | x |  |  |  |
| LHFPL6 | 2 |  |  |  |  |  |  |  |  |  |  | x | x |  |  |  |
| ERG | 2 |  |  |  |  |  |  |  |  |  |  | x | x |  |  |  |
| LTBP1 | 2 |  |  |  |  |  |  |  |  |  |  | x | x |  |  |  |
| ADGRB3 | 2 |  |  |  |  |  |  |  |  |  |  | x | x |  |  |  |
| RAPGEF4 | 2 |  |  |  |  |  |  |  |  |  |  | x | x |  |  |  |
| Only markers with a minimum of 2 reports in recent literature are listed. Location in paper listed in the next table. n obs = number of hits in the database search | | | | | | | | | | | | | | | | |

| **Supplementary table 1. References** | **Original data** |
| --- | --- |
| Hansen J, Sealfon R, Menon R, et al.  A reference tissue atlas for the human kidney.  Sci Adv. 2022;8(23):eabn4965. doi:10.1126/sciadv.abn4965 | Supplementary Table 13, Supplementary Table 5 |
| He, B., Chen, P., Zambrano, S. et al.  Single-cell RNA sequencing reveals the mesangial identity and species diversity of glomerular cell transcriptomes.  Nat Commun 12, 2141 (2021). https://doi.org/10.1038/s41467-021-22331-9 | Figure 4B and main text |
| Kuppe, C., Ibrahim, M.M., Kranz, J. et al.  Decoding myofibroblast origins in human kidney fibrosis.  Nature 589, 281–286 (2021). https://doi.org/10.1038/s41586-020-2941-1 | Supplementary Data 2 |
| Lake, B.B., Chen, S., Hoshi, M. et al.  A single-nucleus RNA-sequencing pipeline to decipher the molecular anatomy and pathophysiology of human kidneys.  Nat Commun 10, 2832 (2019). https://doi.org/10.1038/s41467-019-10861-2 | Supplementary Data 5 Supplementary Data 7 |
| Lake, B.B., Menon, R., Winfree, S. et al.  An atlas of healthy and injured cell states and niches in the human kidney.  Nature 619, 585–594 (2023). https://doi.org/10.1038/s41586-023-05769-3 | Supplementary Table 5 |
| Liu P, Lassén E, Nair V, et al.  Transcriptomic and Proteomic Profiling Provides Insight into Mesangial Cell Function in IgA Nephropathy.  J Am Soc Nephrol. 2017;28(10):2961-2972. doi:10.1681/ASN.2016101103 | Supplementary Table 2 |
| Marshall JL, Noel T, Wang QS, et al.  High-resolution Slide-seqV2 spatial transcriptomics enables discovery of disease-specific cell neighborhoods and pathways.  iScience. 2022;25(4):104097. Published 2022 Mar 16. doi:10.1016/j.isci.2022.104097 | Supplementary figure 1, panel A |
| Menon, R., Otto, E. A., Hoover, P., et al.  Nephrotic Syndrome Study Network (NEPTUNE) (2020). Single cell transcriptomics identifies focal segmental glomerulosclerosis remission endothelial biomarker.  JCI insight, 5(6), e133267. https://doi.org/10.1172/jci.insight.133267 | Supplementary Table 1 |
| Muto, Y., Wilson, P.C., Ledru, N. et al.  Single cell transcriptional and chromatin accessibility profiling redefine cellular heterogeneity in the adult human kidney.  Nat Commun 12, 2190 (2021). https://doi.org/10.1038/s41467-021-22368-w | Supplementary data 1 |
| Wilson, P.C., Muto, Y., Wu, H. et al.  Multimodal single cell sequencing implicates chromatin accessibility and genetic background in diabetic kidney disease progression.  Nat Commun 13, 5253 (2022). https://doi.org/10.1038/s41467-022-32972-z | Supplementary data 5 |
| Young MD, Mitchell TJ, Vieira Braga FA, et al.  Single-cell transcriptomes from human kidneys reveal the cellular identity of renal tumors.  Science. 2018;361(6402):594-599. doi:10.1126/science.aat1699 | Supplementary Table 2 Supplementary Table 4 |
| Zhang Y, Narayanan SP, Mannan R, et al.  Single-cell analyses of renal cell cancers reveal insights into tumor microenvironment, cell of origin, and therapy response.  Proc Natl Acad Sci U S A. 2021;118(24):e2103240118. doi:10.1073/pnas.2103240118 | Dataset Supplement 2A |
