# Supplementary material for: Comparative analysis of commercial human primary mesangial cell, implications for experimental design
Source: BMC Nephrol. 2025 Sep 29;26:539. doi: 10.1186/s12882-025-04444-1 (PMC12482395; doi:10.1186/s12882-025-04444-1)
Supplement: Supplementary file 2 — Supplementary Material 2 [file 12882_2025_4444_MOESM2_ESM.pdf]

Uncropped gel, Figure 1C

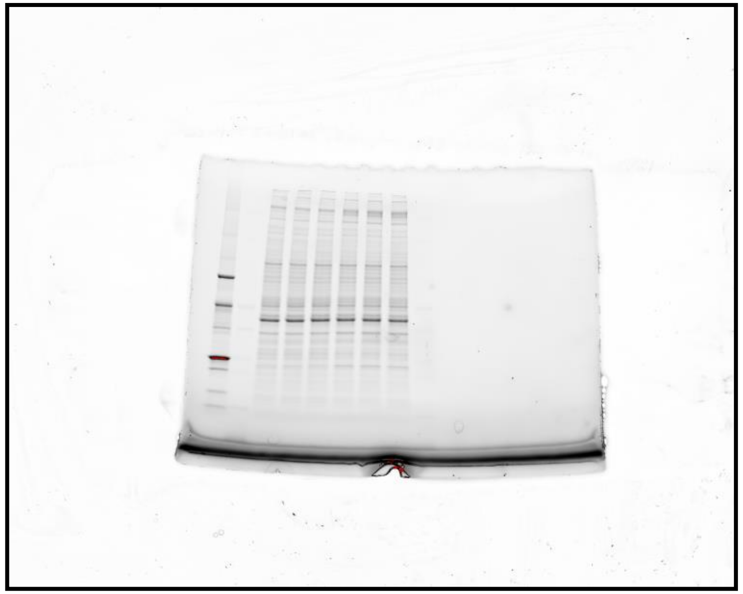

Stain free gel

Uncropped blot, Figure 1C

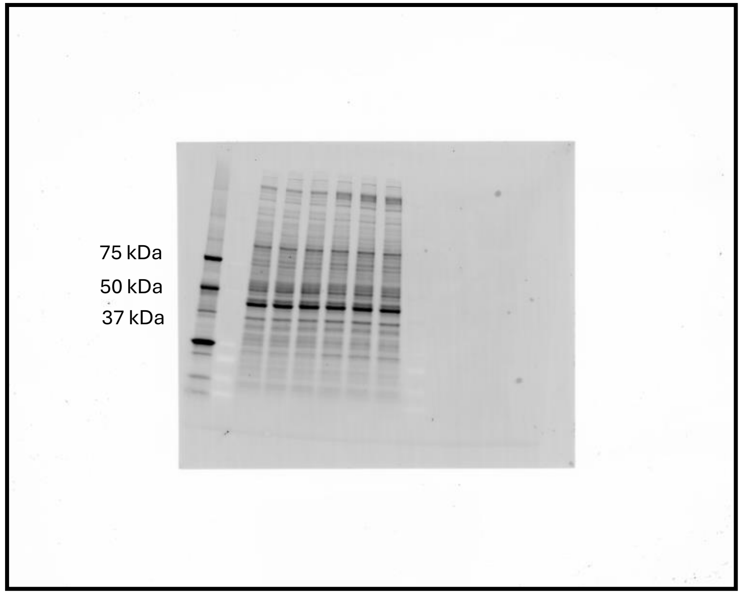

Stain free blot (total lane protein content)

Upper part of blot (>50kDa)

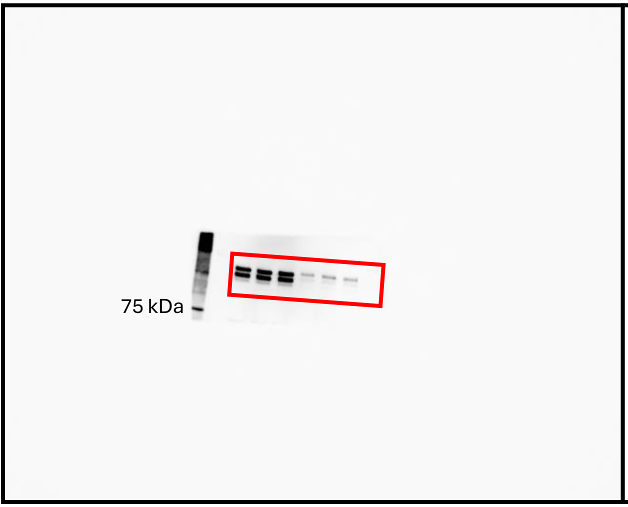

PDGFRA blot

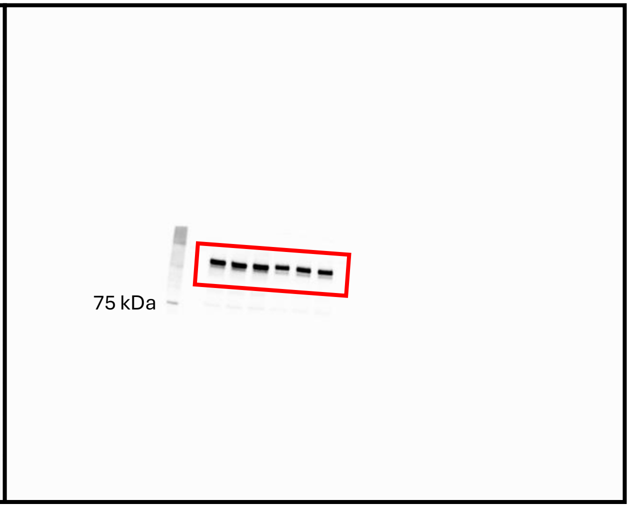

PDGFRB blot

Lower part of blot (<50 kDa)

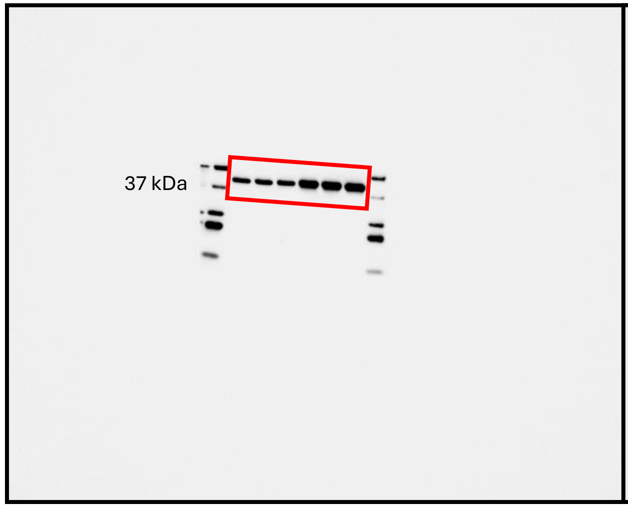

ACTA2 blot

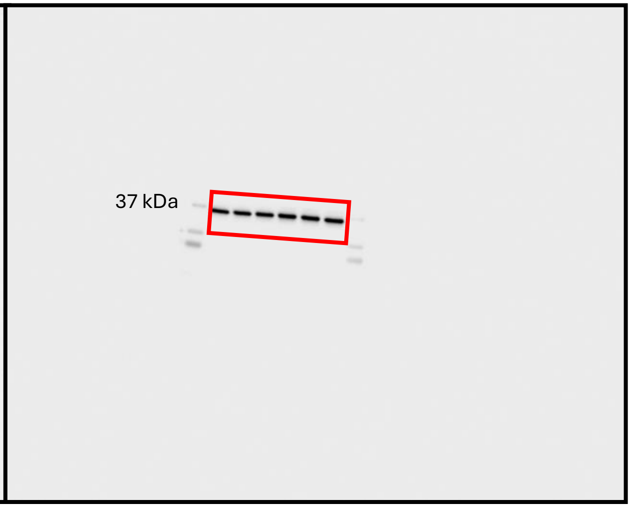

GAPDH blot

Uncropped gel 1, Figure 4F

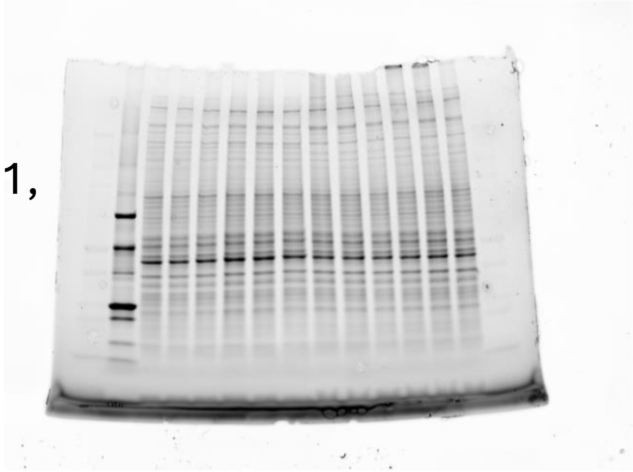

Uncropped gel 2, Figure 4F

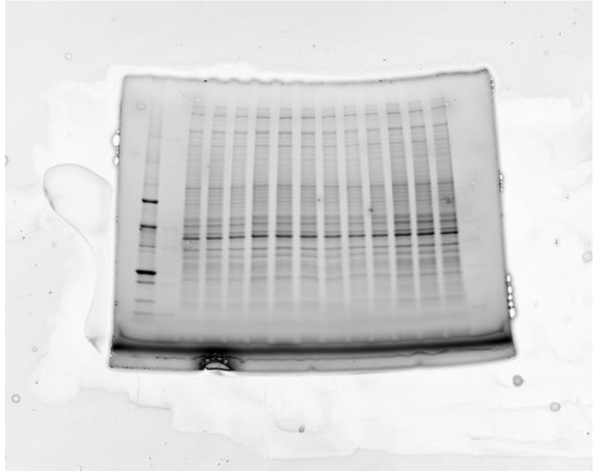

Uncropped blot 1, Figure 4F

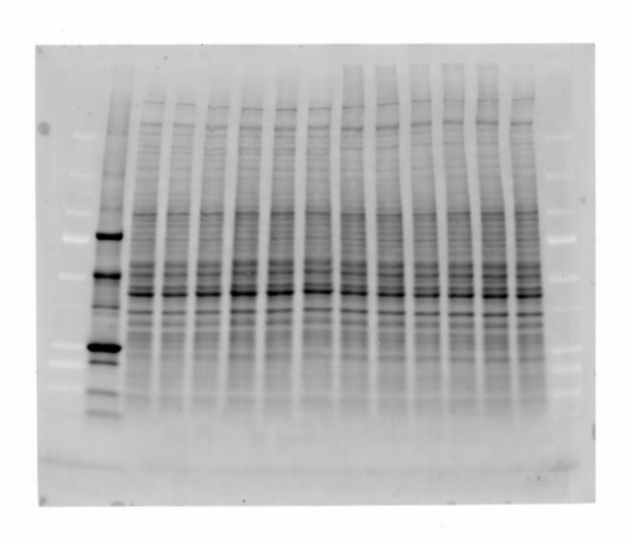

Uncropped blot 2, Figure 4F

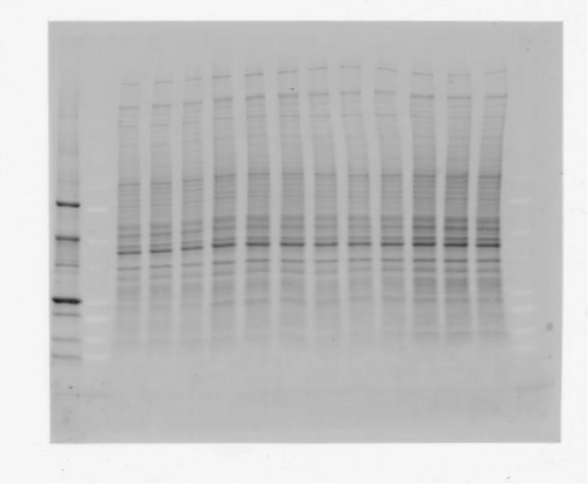

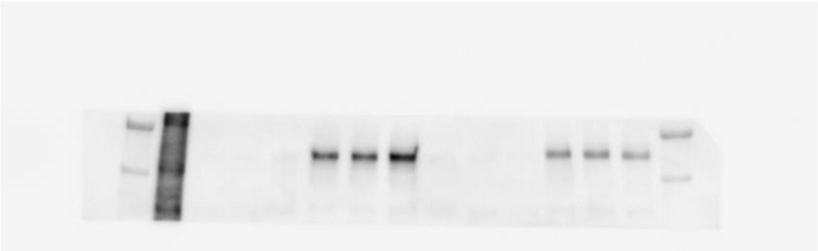

PDGFRB T751 blot

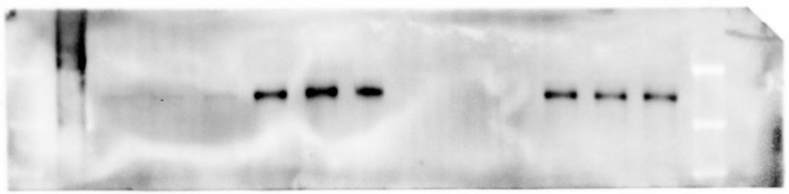

PDGFRB T1009 blot
