# Supplementary figures and images for: Comparative analysis of commercial human primary mesangial cell, implications for experimental design
Source: BMC Nephrol. 2025 Sep 29;26:539. doi: 10.1186/s12882-025-04444-1 (PMC12482395; doi:10.1186/s12882-025-04444-1)

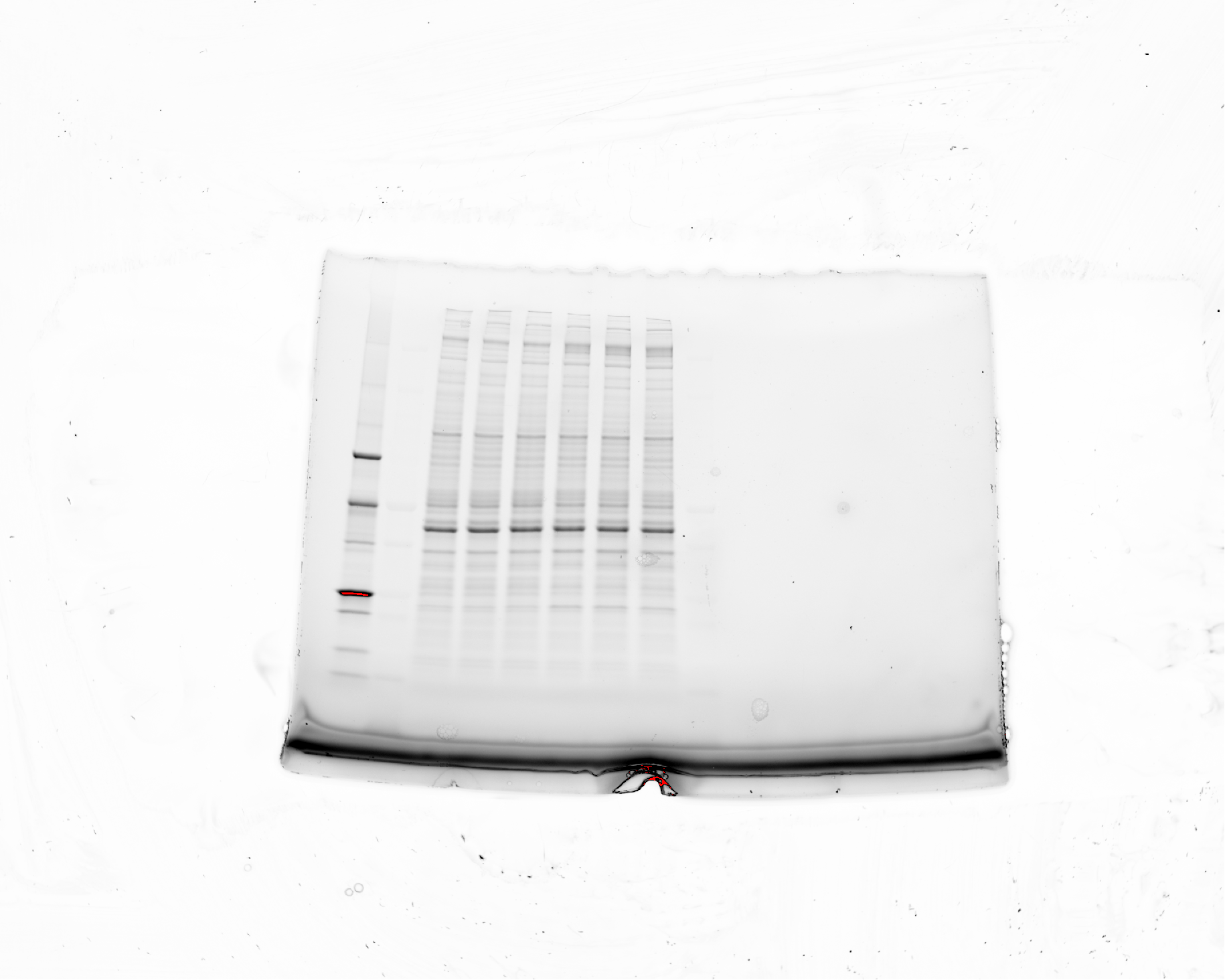

Supplement: Supplementary file 3 — Supplementary Material 3 [file 12882_2025_4444_MOESM3_ESM.tif]

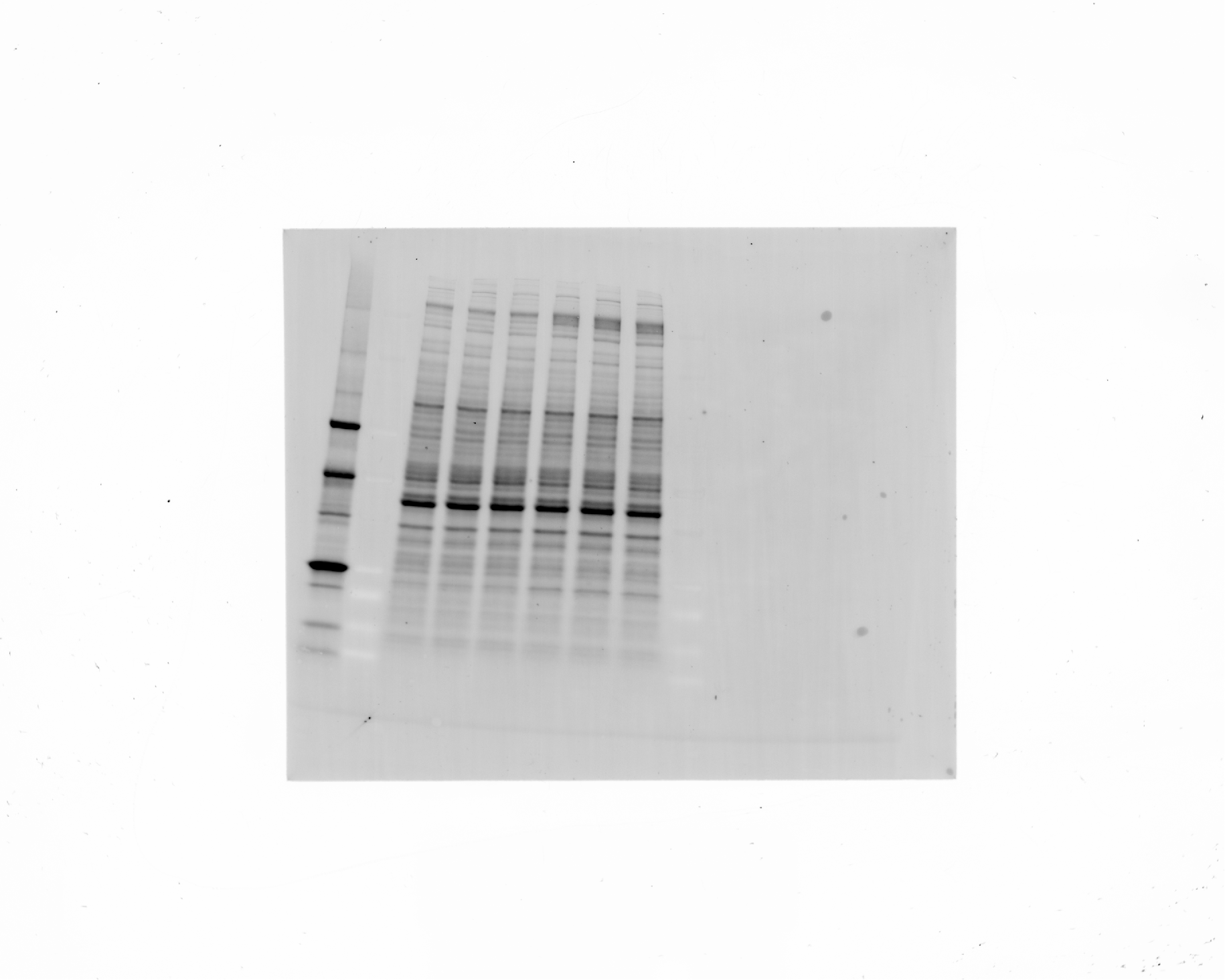

Supplement: Supplementary file 5 — Supplementary Material 5 [file 12882_2025_4444_MOESM5_ESM.tif]

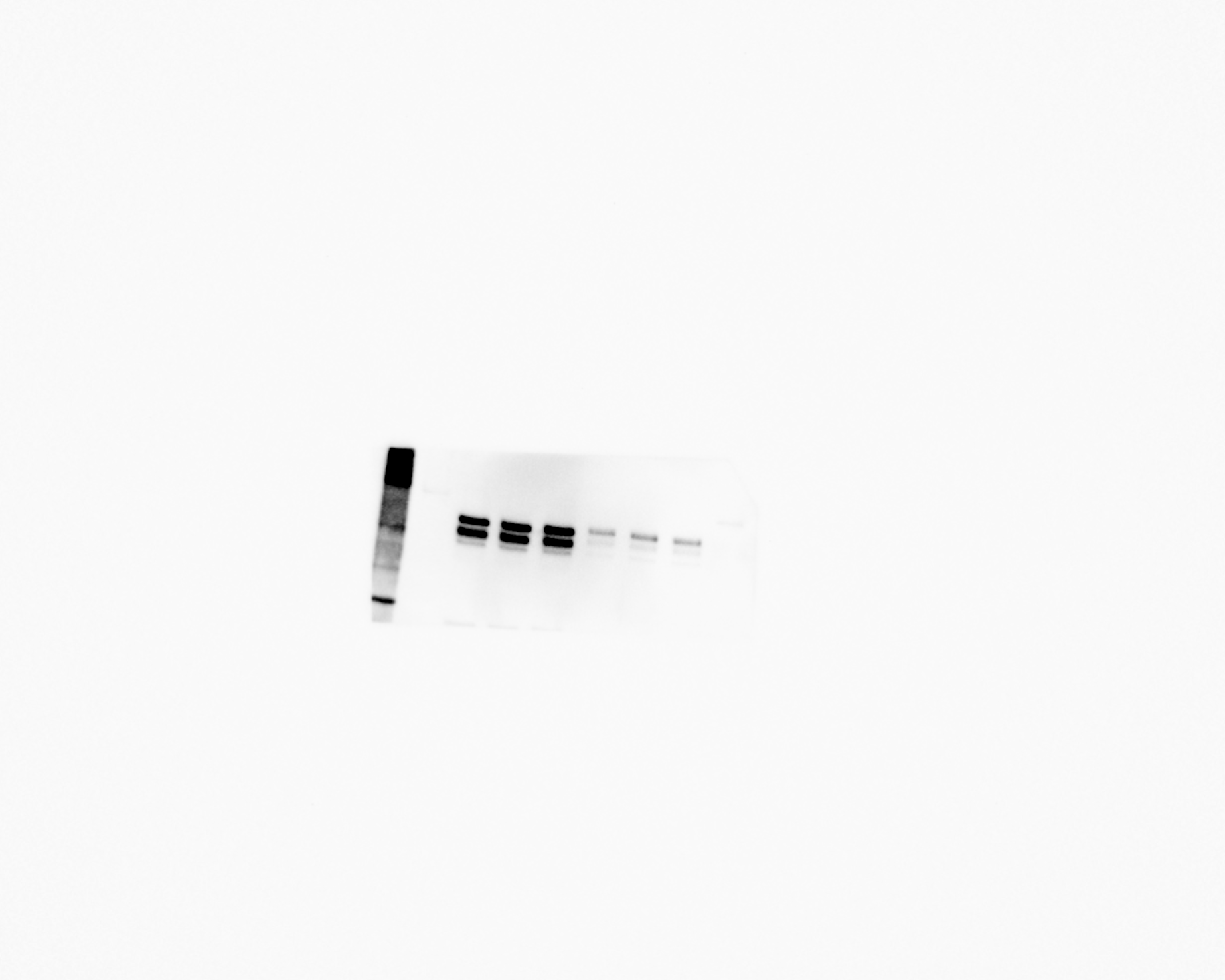

Supplement: Supplementary file 6 — Supplementary Material 6 [file 12882_2025_4444_MOESM6_ESM.tif]

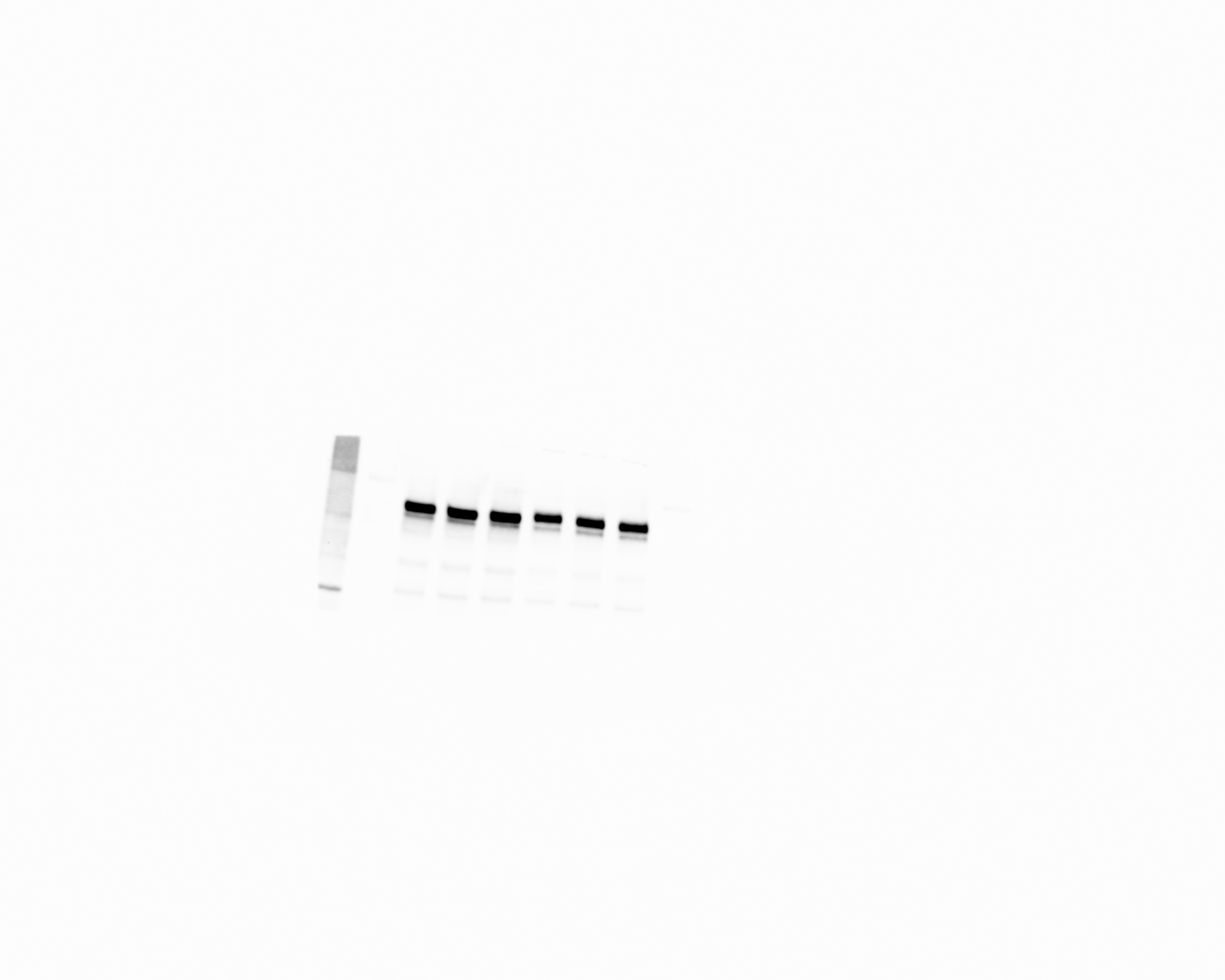

Supplement: Supplementary file 7 — Supplementary Material 7 [file 12882_2025_4444_MOESM7_ESM.tif]

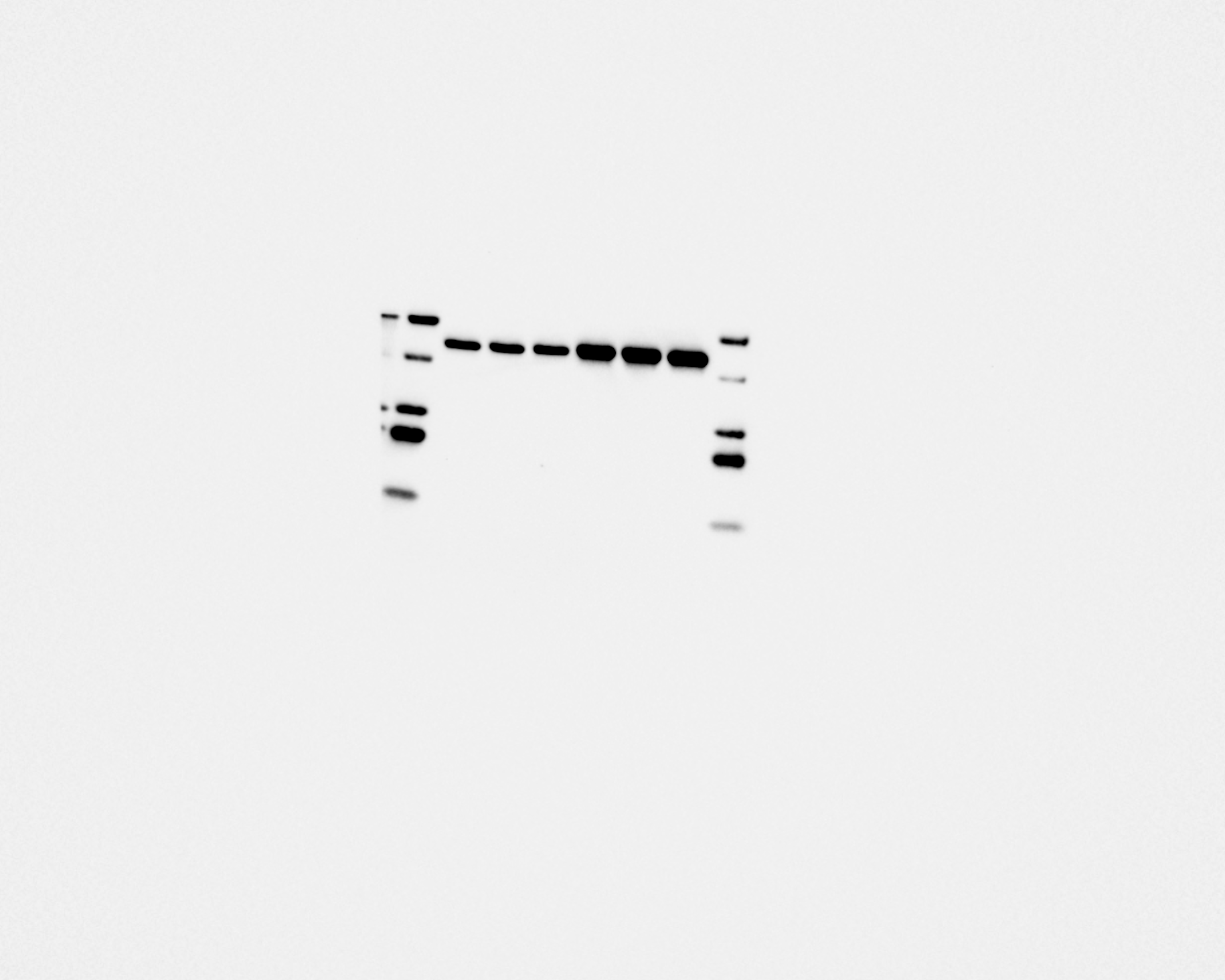

Supplement: Supplementary file 8 — Supplementary Material 8 [file 12882_2025_4444_MOESM8_ESM.tif]

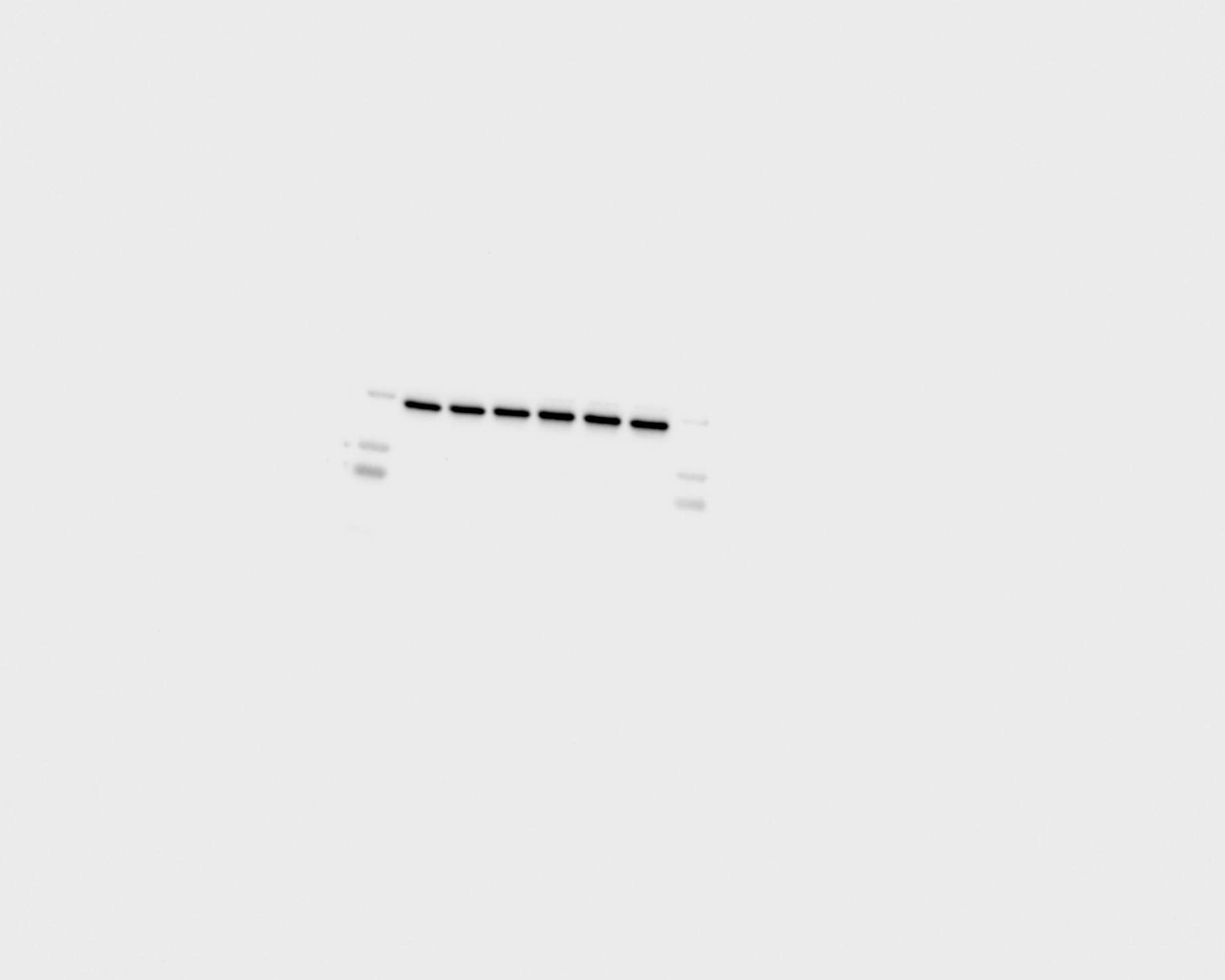

Supplement: Supplementary file 9 — Supplementary Material 9 [file 12882_2025_4444_MOESM9_ESM.tif]

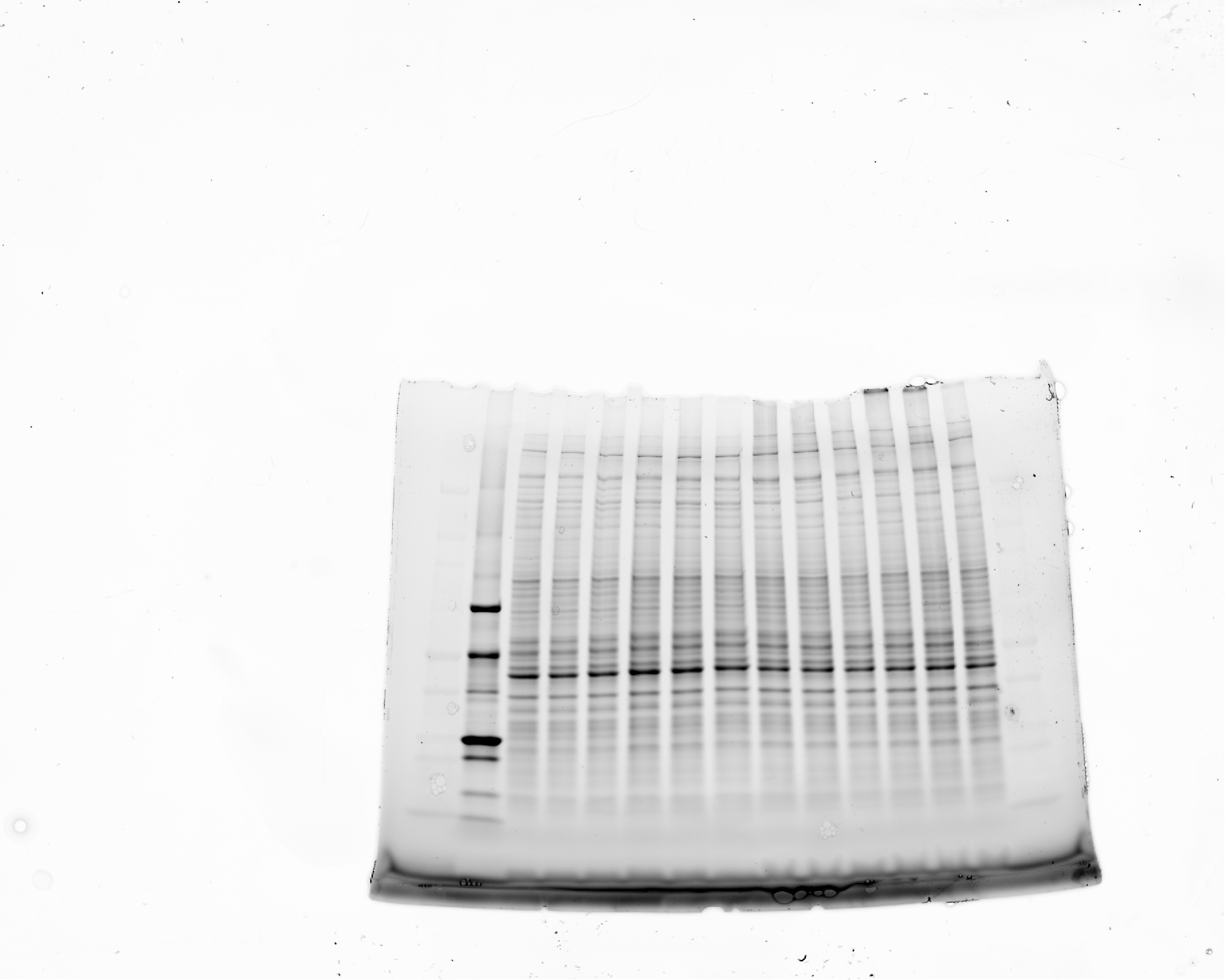

Supplement: Supplementary file 10 — Supplementary Material 10 [file 12882_2025_4444_MOESM10_ESM.tif]

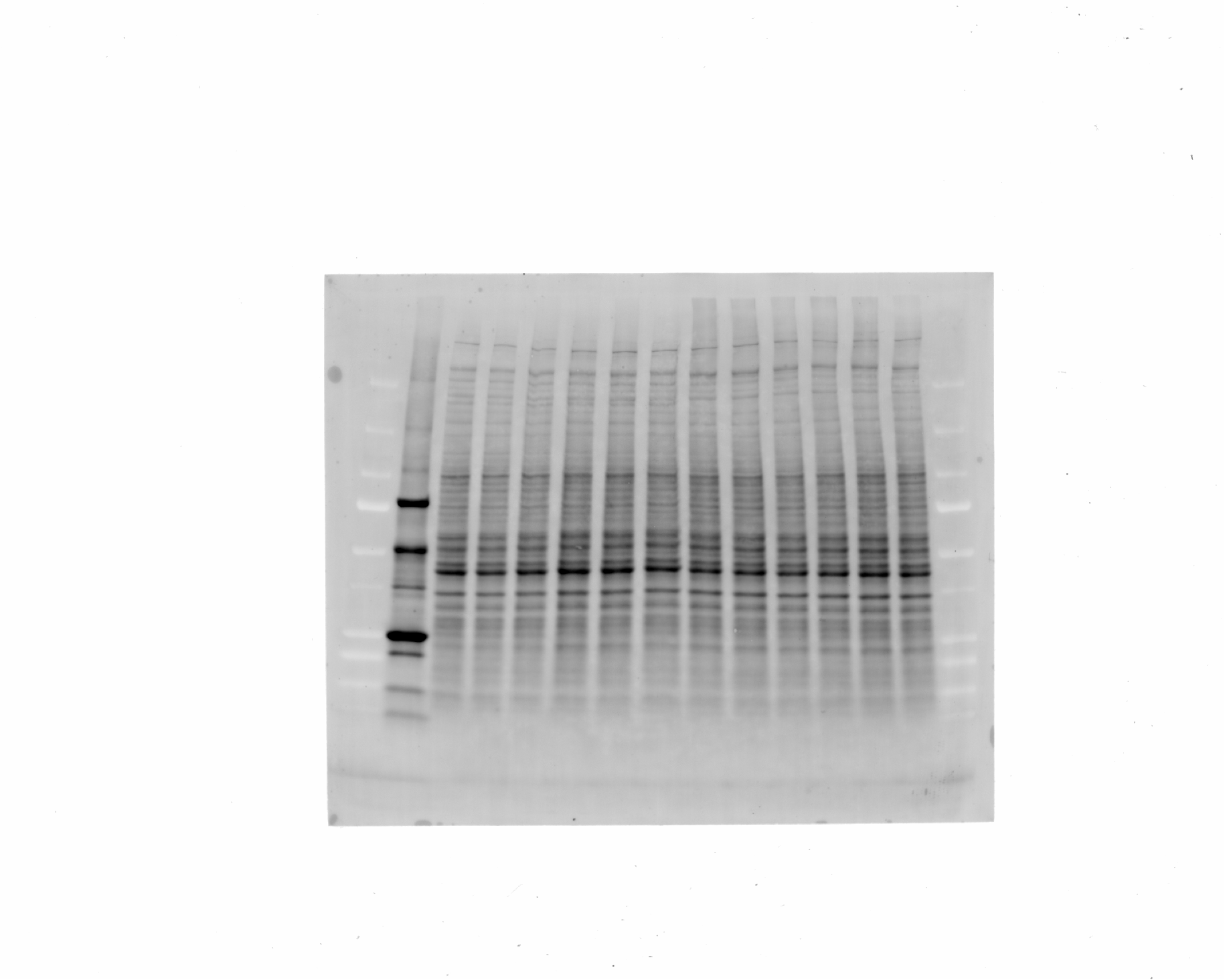

Supplement: Supplementary file 11 — Supplementary Material 11 [file 12882_2025_4444_MOESM11_ESM.tif]

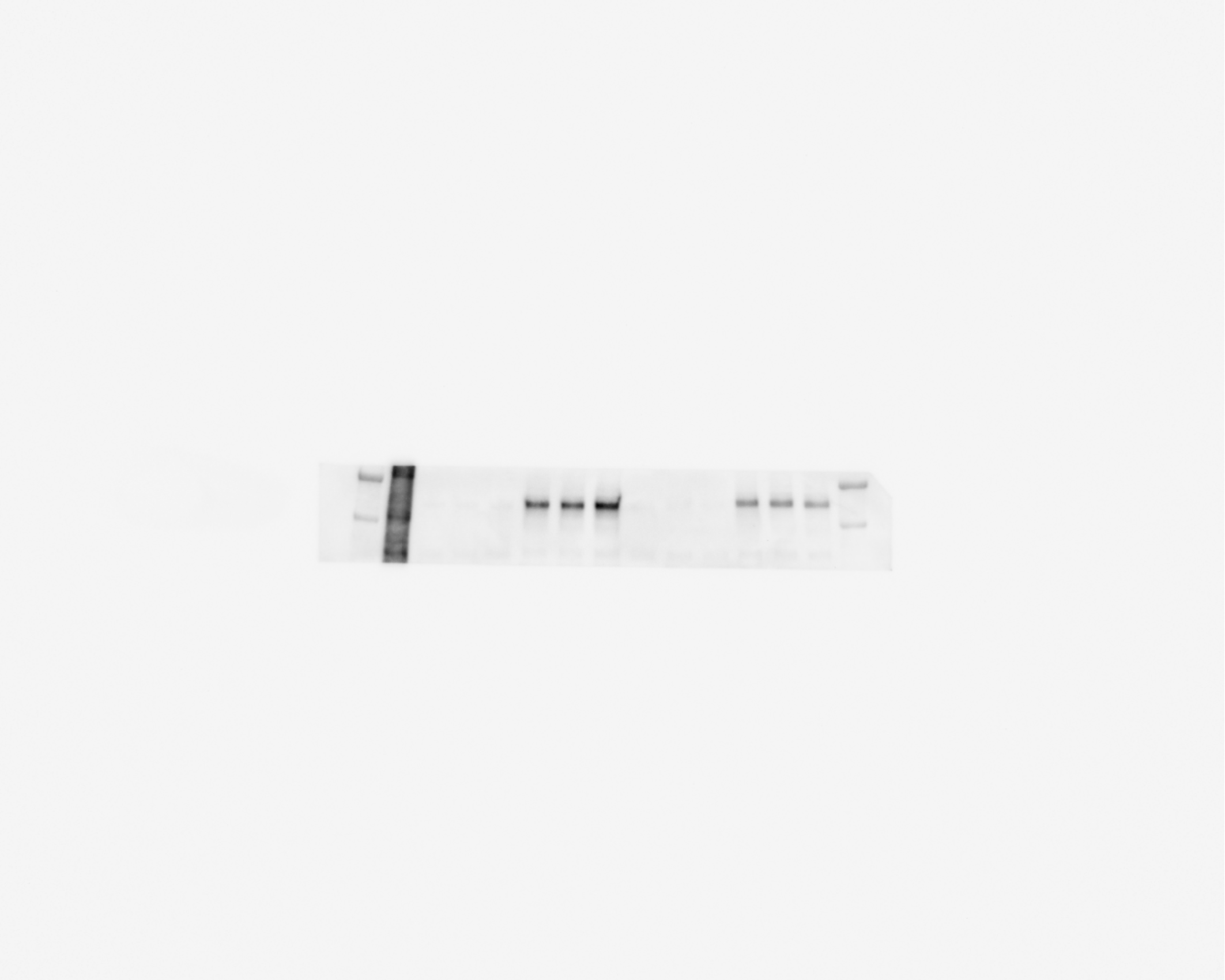

Supplement: Supplementary file 12 — Supplementary Material 12 [file 12882_2025_4444_MOESM12_ESM.tif]

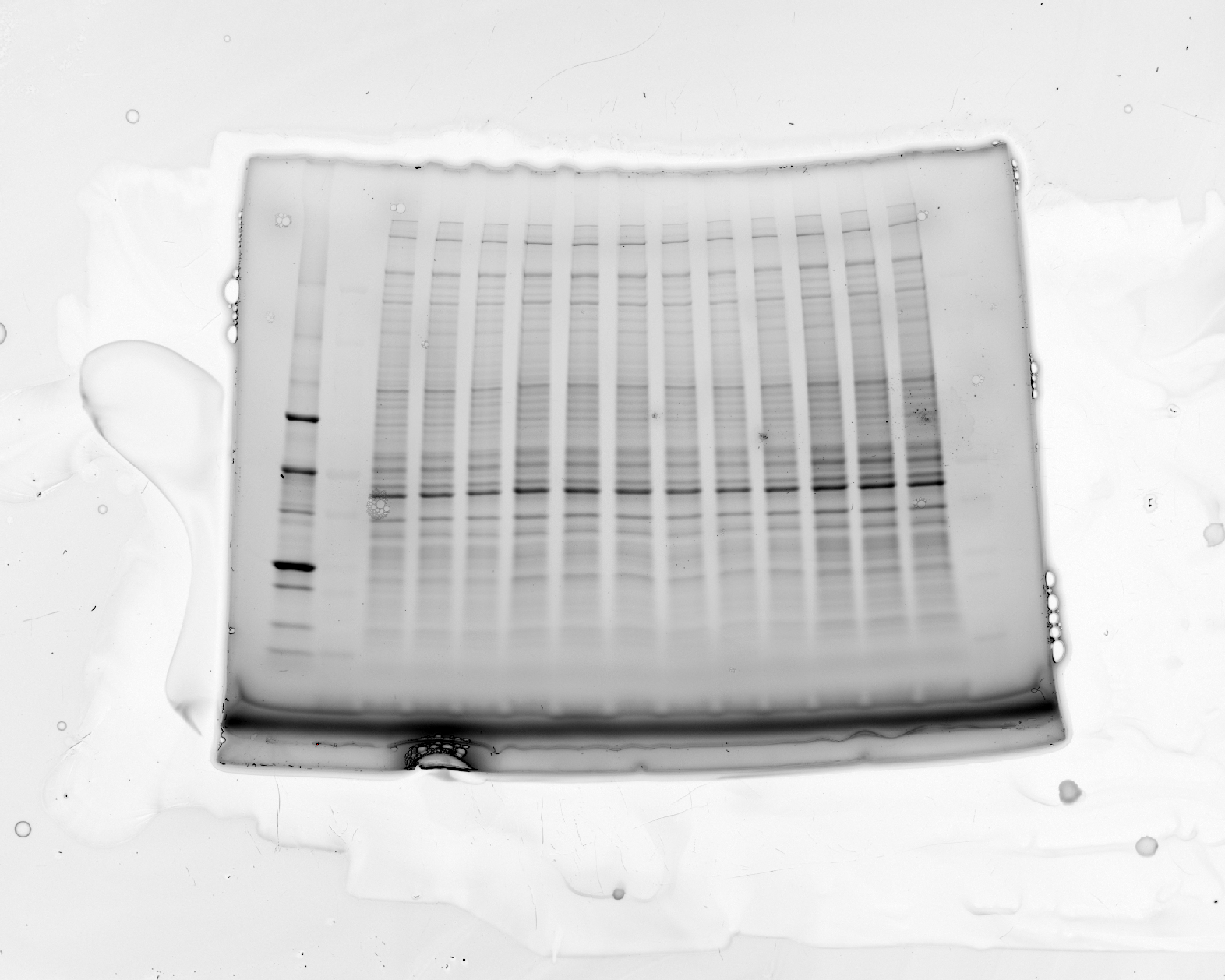

Supplement: Supplementary file 13 — Supplementary Material 13 [file 12882_2025_4444_MOESM13_ESM.tif]

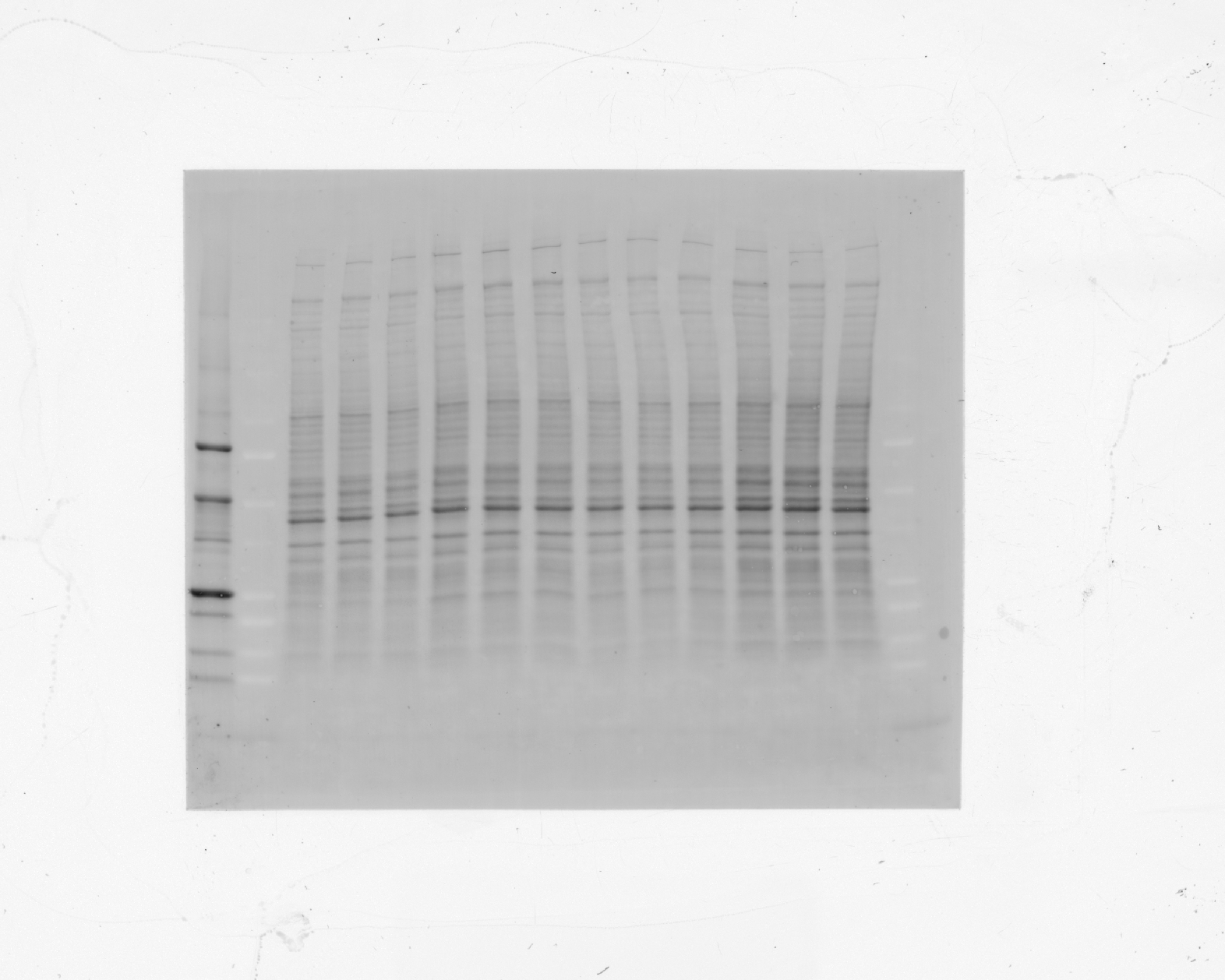

Supplement: Supplementary file 14 — Supplementary Material 14 [file 12882_2025_4444_MOESM14_ESM.tif]

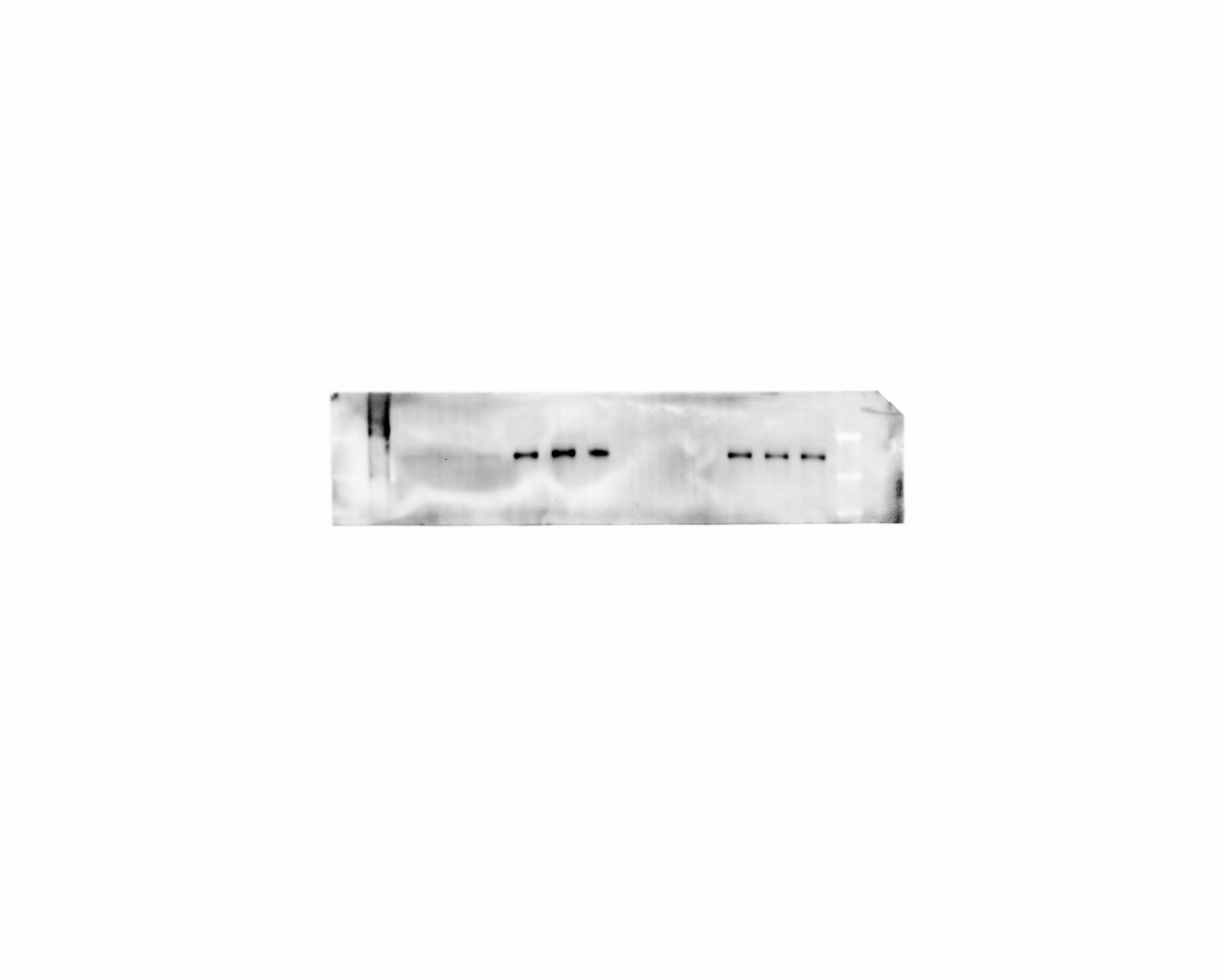

Supplement: Supplementary file 15 — Supplementary Material 15 [file 12882_2025_4444_MOESM15_ESM.tif]
