# Supplementary material for: Comparative analysis of commercial human primary mesangial cell, implications for experimental design
Source: BMC Nephrol. 2025 Sep 29;26:539. doi: 10.1186/s12882-025-04444-1 (PMC12482395; doi:10.1186/s12882-025-04444-1)
Supplement: Supplementary file 4 — Supplementary Material 4 [file 12882_2025_4444_MOESM4_ESM.pptx]

## Slide 1
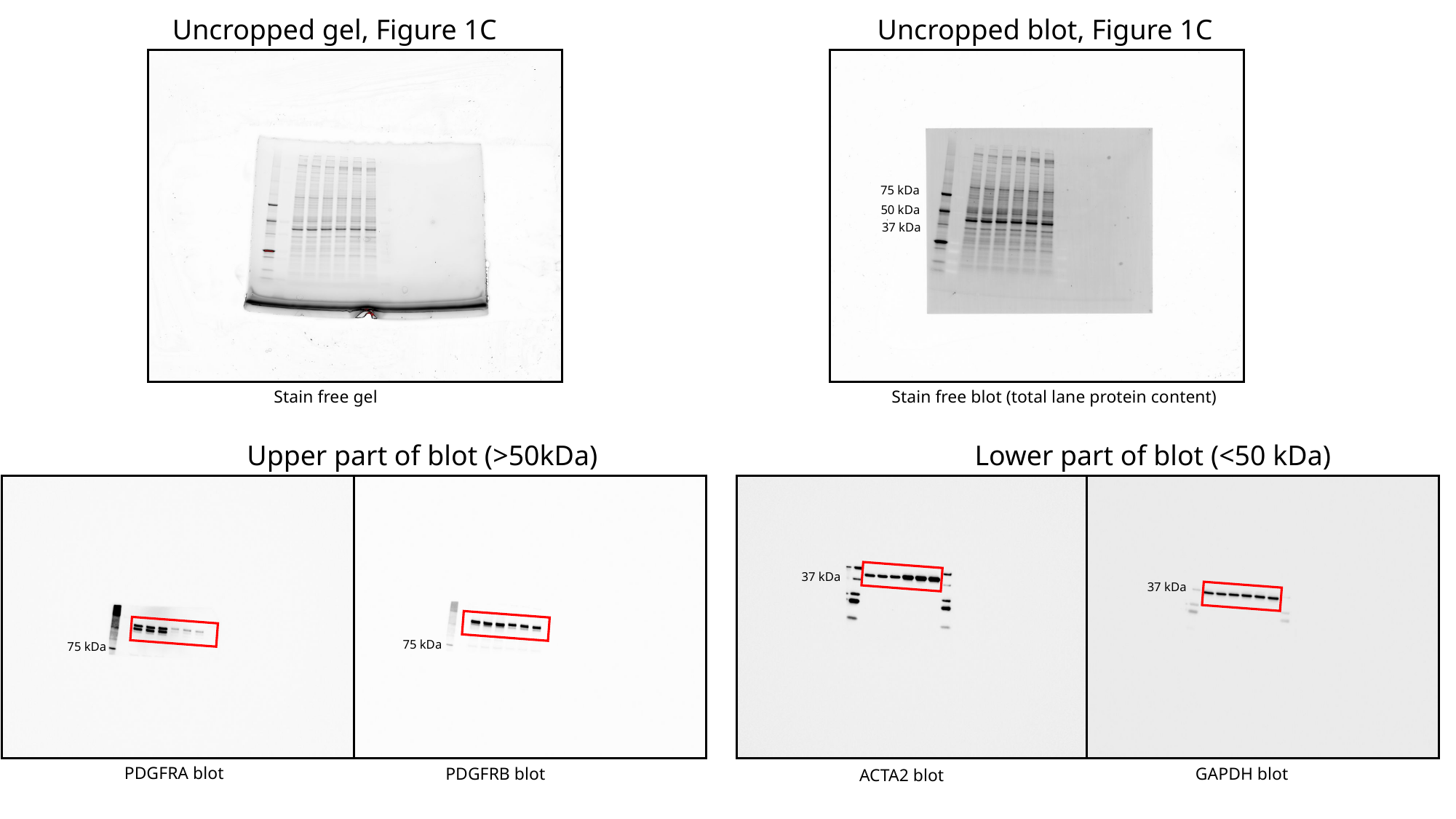

Uncropped gel, Figure 1C
Uncropped blot, Figure 1C
75 kDa
50 kDa
37 kDa
Stain free gel
Stain free blot (total lane protein content)
Upper part of blot (>50kDa)
Lower part of blot (<50 kDa)
37 kDa
37 kDa
75 kDa
75 kDa
PDGFRA blot
GAPDH blot
PDGFRB blot
ACTA2 blot

## Slide 2
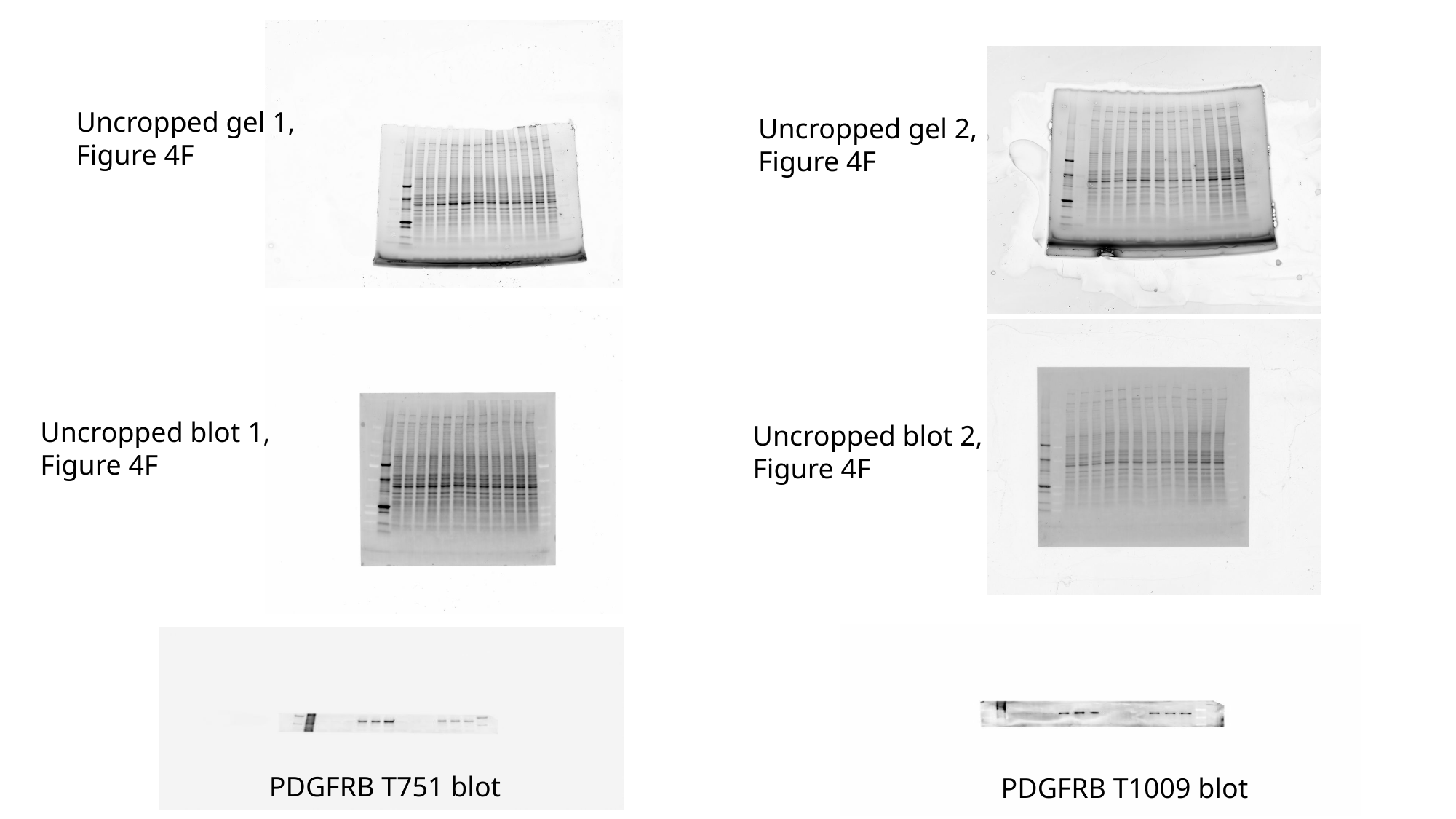

Uncropped gel 1,
Figure 4F
Uncropped gel 2,
Figure 4F
Uncropped blot 1,
Figure 4F
Uncropped blot 2,
Figure 4F
PDGFRB T751 blot
PDGFRB T1009 blot
